# Phosphatase Shp2 regulates biogenesis of small extracellular vesicles by dephosphorylating Syntenin

**DOI:** 10.1002/jev2.12078

**Published:** 2021-03-10

**Authors:** Yuefei Zhang, Yiqing Li, Pan Liu, Dacheng Gong, Hui Zhou, Wenjuan Li, Huilun Zhang, Wenfang Zheng, Jiaqi Xu, Hongqiang Cheng, Xue Zhang, Yuehai Ke

**Affiliations:** ^1^ Department of Pathology and Pathophysiology and Department of Respiratory Medicine at Sir Run Run Shaw Hospital Zhejiang University School of Medicine Hangzhou Zhejiang 310058 China; ^2^ Zhejiang Laboratory for Systems and Precision Medicine Zhejiang University Medical Center Hangzhou 311121 China; ^3^ Department of Pathology and Pathophysiology and Department of Cardiology at Sir Run Run Shaw Hospital Zhejiang University School of Medicine Hangzhou Zhejiang 310058 China; ^4^ Department of Obstetrics and Gynecology Women's hospital Zhejiang University School of Medicine Hangzhou Zhejiang 310058 China; ^5^ Department of Gastroenterology Sir Run Run Shaw Hospital Zhejiang University School of Medicine Hangzhou Zhejiang 310058 China; ^6^ Department of Pathology Sir Run Run Shaw Hospital Zhejiang University School of Medicine Hangzhou Zhejiang 310058 China

**Keywords:** biogenesis, crosstalk, dephosphorylation, epithelial cells, macrophages, phosphatase, Shp2, small extracellular vesicles, Syntenin

## Abstract

As novel mediators of cell‐to‐cell signalling, small extracellular vesicles (sEVs) play a critical role in physiological and pathophysiological processes. To date, the molecular mechanisms that support sEV generation are incompletely understood. Many kinases are reported for their roles in sEV generation or composition, whereas the involvement of phosphatases remains largely unexplored. Here we reveal that pharmacological inhibition and shRNA‐mediated down‐regulation of tyrosine phosphatase Shp2 significantly increases the formation of sEVs. By Co‐immunoprecipitation (Co‐IP) and in vitro dephosphorylation assays, we identified that Shp2 negatively controlled sEV biogenesis by directly dephosphorylating tyrosine 46 of Syntenin, which has been reported as a molecular switch in sEV biogenesis. More importantly, Shp2 dysfunction led to enhanced epithelial sEV generation in vitro and in vivo. The increase of epithelial sEVs caused by shRNA‐mediated down‐regulation of Shp2 promoted macrophage activation, resulting in strengthened inflammation. Our findings highlight the role of Shp2 in regulating sEV‐mediated epithelial‐macrophage crosstalk by controlling sEV biogenesis through dephosphorylation of Syntenin Y46. The present study determines the strengthened inflammatory characteristics of alveolar macrophages elicited by epithelial sEVs transferred intercellularly. These findings provide a basis for understanding the mechanism of sEV formation and relevant function in epithelial‐macrophage crosstalk.

## 1 INTRODUCTION

Small extracellular vesicles (sEVs), typically secreted by the exocytosis of multivesicular bodies (MVBs), are nanovesicles (< 200 nm diameter) present in various body and interstitial fluids (Valcz et al., 2019; Witwer & Théry, 2019). sEVs mediate intercellular communication by acting as vehicles containing proteins, lipids, DNA and mRNA (Kourembanas, 2015). Precise regulation of sEV formation has a critical role in cell‐to‐cell crosstalk.

The generation of sEVs originates from the endosome pathway. MVBs are formed by in‐budding of early endosomes, resulting in accumulated intraluminal vesicles (ILVs). The ILVs then fuse with the plasma membrane and are released by the MVBs. sEVs are nano‐scaled ILVs secreted to extracellular environment (Latifkar et al., 2019). sEVs act as messengers for cell‐to‐cell communication and further affect the signalling pathways of acceptor cells, thereby regulating their function (Théry, 2011). In recent years, despite increasing researches on sEVs, the molecular mechanisms regulating sEV production are still unclear. A number of protein complexes are involved in the formation of ILVs in MVBs, namely the biogenesis mechanism of sEVs. These mechanisms mainly include ESCRT‐dependent pathways and ESCRT‐independent pathways. In addition, these two pathways are not entirely separated as they coexist and jointly mediate the formation of sEVs in MVBs. Thus, they work synergistically to generate different subpopulations of sEVs (Baietti et al., 2012).

Syntenin‐Syndecan‐ALIX plays a key role in the biogenesis of sEVs. Syntenin–Syndecan complexes are linked to the ESCRT machinery via ALIX, thus leading to membrane budding and scission at the MVBs (Baietti et al., 2012). It is an intermediate route in which some ESCRT components are involved. Syndecan is the interacting protein of heparan sulfate on the cell surface broadly present in sEVs (Roucourt et al., 2015). Syndecan is packaged into sEVs through the specific adaptor protein Syntenin. Meanwhile, Syntenin interacts with ALIX to sort Syndecan‐mediated cargo into sEVs (Baietti et al., 2012). In addition, heparanase influences the sEV generation regulated by Syndecan–Syntenin–ALIX (Roucourt et al., 2015

). The effect of heparanase on sEV formation is dependent on the interaction between Syntenin and ALIX. As known regulators, small GTPase ADP ribosylation factor 6 (ARF6) and its effector phospholipase D2 (PLD2) affect the biogenesis of Syntenin‐mediated sEVs by controlling the ILV budding (Ghossoub et al., 2014). The kinase Src, upstream of ARF6 and PLD2, promotes sEV biogenesis by phosphorylating the tyrosine of Syntenin (Imjeti et al., 2017). Furthermore, the role of Sytenin in sEV formation is complex and needs further investigation.

As enzymes that catalyze protein phosphorylation, kinases have critical effects in the regulation of various signalling pathways, that are widely involved in cell functions and behaviours (Julien et al., 2011; Proud, 2019). Increasing studies find that various kinases are involved in sEV biogenesis by regulating phosphorylation. The tyrosine kinase Hck is activated by HIV and promotes the release of proinflammatory sEVs containing ADAM protease in myeloid cells and liver cells (Lee et al., 2018). Pyruvate kinase PKM2 enhances the release of tumour cell sEVs by phosphorylating SNAP‐23 (Wei et al., 2017). Protein phosphorylation is under strict regulation and governed by the balanced actions of kinases and phosphatases. The dephosphorylated protein loses its previous function upon removal of the phosphate group. It is noteworthy that the regulation of sEVs by phosphatases remains limited investigation when compared with kinases. Studying phosphatase‐mediated dephosphorylation will help to further explain the mechanism of sEV production.

Src homology 2‐containing protein tyrosine phosphatase 2 (Shp2) is a type of non‐receptor tyrosine phosphatase widely expressed in the cytoplasm. It is composed of two SH2 domains at the N‐terminus and a PTP domain at the C‐terminus. Shp2 is involved in controlling various cell function and disease processes through dephosphorylation (Tajan et al., 2015). Moreover, Shp2 regulates a variety of pathological processes of lung, and plays an important regulatory role in the alveolar microenvironment. Loss of Shp2 in lung epithelial cells alleviates smoke‐mediated lung inflammation (Li et al., 2012). Deletion of neutrophil Shp2 relieves acute lung injury induced by LPS (Zhang et al., 2016). Till now, we are far from sufficient understanding of intercellular sEV transfer, because of limited cellular and animal models (Fujita et al., 2015). sEVs play an important role in the intercellular communication of lung microenvironment, primarily orchestrated by lung epithelial cells. Repeated damage on lung epithelial cells triggers adaptive signalling pathway to maintain physiological homeostasis (Matthay et al., 2019). Functional phenotypes of alveolar macrophages are modulated by corresponding sEVs derived from changed microenvironment (Raposo & Stoorvogel, 2013). Analyzing the role of epithelial Shp2 in regulating the alveolar microenvironment will strengthen the understanding of pulmonary pathogenesis.

**STATEMENT OF SIGNIFICANCE**

Our findings reveal the role of tyrosine phosphatase Shp2 in negatively regulating sEV biogenesis through dephosphorylation of Syntenin. This mechanism mediates the epithelial‐macrophage crosstalk in inflammatory microenvironment. These findings provide a basis for understanding the mechanism of sEV formation and relevant function in epithelial‐macrophage crosstalk.

In the present study, our investigations reveal that pharmacologic inhibition and shRNA‐mediated down‐regulation of tyrosine phosphatase Shp2 increases the sEV biogenesis both in vitro and in vivo. Indicated by nanoparticle tracking analysis, Shp2 pharmacologic inhibition and shRNA‐mediated down‐regulation enhanced sEV release in various cells. Supported by transmission electron microscopy (TEM) analysis, in vivo evidence showed accumulated ILVs in MVBs by Shp2 depletion. The tyrosine phosphorylation of Syntenin, reported to promote ILV generation in MVBs, was significantly upregulated when Shp2 was depleted. With the help of Co‐IP and dephosphorylation assay, we found that Shp2 negatively controlled sEV biogenesis by directly dephosphorylating Syntenin Y46. Meanwhile, Shp2 stable knockdown (KD) created no significant changes in components and subtypes of epithelial sEVs, indicated by the data of mass spectrometry (MS) and nanoscale flow cytometry. Analyzed by in vitro donor–acceptor sEV transfer system, loss of Shp2 enhanced sEV‐mediated epithelial‐macrophage crosstalk. The in vivo loss of epithelial Shp2 induced increased epithelial sEVs and resulted in enhanced macrophage activation and lung inflammation. Here, we show that Shp2 is a regulator of syntenin‐dependent endosomal trafficking via its phosphatase activity. These results reveal that shRNA‐mediated down‐regulation of Shp2 induces release of epithelial sEVs, leading to macrophage activation and lung inflammation.

## 2 MATERIAL AND METHODS

### 2.1 Expression vectors and reagents

RAB5(Q79L)‐GFP was constructed to induce enlarged endosomes. To construct Shp2‐Myc, Syntenin‐mCherry, Syntenin‐Flag, CD9‐Flag, ALIX‐Flag, Flotillin 1‐Flag, YKT6‐Flag, the according human full‐length sequences were amplified and cloned into the PXJ40 vectors. To produce Syntenin‐GST, Syntenin‐Y46F‐GST, Src‐GST, Shp2 FL‐GST, Shp2 PTP‐GST, the according human sequences were amplified and cloned into the Pcold‐GST plasmids. Shp2‐E76V‐Myc, Syntenin‐Y46E‐mCherry, Syntenin‐Y46F‐mCherry were generated by point mutation in cDNA encoding full‐length sequences cloned in the PXJ40 expression vector. All constructs were controlled by sequencing.

Antibodies against Shp2 (ab131541), Syntenin (ab133267, ab19903), CD9 (ab92726), ALIX (ab186429), TSG101 (ab125011), HSC70 (ab51052), Annxein A1 (ab214486), Syndecan‐1 (ab128936), GM130 (ab187514), Calnexin (ab22595), GRP94 (ab238126), CD326 (ab213500), iNOS (ab178945) and CD68 (ab955) were from Abcam; Antibody against Flag (TA50011‐100) and Myc (TA150121) were from OriGene; Antibodies against Flotillin 1 (sc‐74566), YKT6 (sc‐365732), CHMP4A (sc‐514869), SPC(sc‐13979) and Hrs (sc‐271455) were from Santa Cruz Biotechnology; Antibodies against P‐Tyr (8954), P‐Src 416 (6943) and Src (2109) were from Cell Signaling Technology. β‐Actin (ET1701‐80) antibody was from HuaBio. For flow cytometric analysis, antibodies against CD9 (11‐0091‐81, eBioscience), CD63 (17‐0631‐80, eBioscience) and CD81 (104905, BioLegend) were used.

RNAi targeting mouse Syntenin and the non‐targeting control RNAi (siScramble) were purchased from General Biol (Anhui) Inc. siSyntenin (5′‐GCAAGGGAUCCGUGAAGUUTT‐3′); siScramble (5′‐UUCUCCGAACGUG

UCACGUTT‐3′); RNAi targeting mouse Shp2: siShp2 #1 (5′‐ GGACAUGAAUAUACCAAUATT‐3′); siShp2 #2 (5′‐ GGAUGGUGUUCCAGGAGAATT‐3′).tr

The inhibitors include CAY10594 (sc‐223874, TargetMol), SHP099 (S8278, Selleck), VO‐Ohpic (CSN18294, CSNpharm), PTP1B‐IN‐2 (CSN23553, CSNpharm), NQ301 (S0141, Selleck), TPI‐1 (S6570, Selleck), Etoposide (S1225, Selleck), PHPS1 (540213, Sigma) and Sodium orthovanadate (SOV, S6508, Sigma).

### 2.2 Cell culture and transfections

MLE‐12 cells were grown in DMEM/F12 (1:1) basic medium (Gibco) supplemented with 2% FBS (ExCell Bio). MCF‐7, 293T and HUVEC‐C cells were grown in DMEM basic medium (Gibco) supplemented with 10% FBS (ExCell Bio). RAW 264.7 cells were grown in DMEM basic medium (Gibco) supplemented with 10% heat inactivated FBS (ExCell Bio). Jurkat cells were grown in RPMI 1640 medium (Gibco) supplemented with 10% FBS (ExCell Bio).

For transient expressions, the cells were transfected using Lipo2000 (Invitrogen) according the manual. For siRNA experiments, cells were treated with 50 nM siRNA and Lipo3000 (Invitrogen); cells were analyzed after 48 or 72 h of RNAi treatment. Bone marrow–derived macrophages (BMDMs) were transfected with siRNA using Lipofectamine RNAiMAX (Invitrogen) according to the standard protocol.

### 2.3 Nanoparticle tracking analysis (NTA)

The number and size of sEVs were directly tracked using the Nanosight NS 500 system (Malvern). In this analysis, particles were automatically tracked and sized based on Brownian motion and the diffusion coefficient. The sEV pellets were resuspended in PBS and further diluted 100‐ to 500‐fold to achieve between 20 and 100 objects per frame. The samples were loaded manually into the sample chamber at ambient temperature. Each sample was measured in in three times with acquisition time of 60 s. The NTA 2.3 software was used for capturing and analyzing the video data.

### 2.4 sEV isolation

For sEV purification experiments, the serum was depleted of sEVs, by centrifugation at 110,000 × *g* for 12 h (Thery et al., 2006). The sEVs in cell supernatant and BALF (Bronchoalveolar lavage fluid) were isolated by four steps at 4°C:(1) 5 min at 500 × *g*, to remove cells; (2) 20 min at 2000 × *g*, to remove cell debris; (3) filtrated through 0.22 μm filters (4)1.5 h at 110,000 × *g*, to pellet sEVs—followed by one wash (by resuspension, centrifugation) in PBS. Particle concentrations were determined by NTA. Total sEV protein was measured by Bradford Quick Start assay (Bio‐Rad).

### 2.5 Knockdown by Lenti‐shRNA

Shp2 knockdown was performed by lentiviral expression of short hairpin RNA (shRNA) targeting human Shp2 (shShp2 #1, 5′‐CGCTAAGAGAACTTAAACTTT‐3′; shShp2 #2, 5′‐AGATGTCATTGAGCTTAAATA‐3′;), and 5′‐ACTACCGTTGTTATAGGTGT‐3′ was used as the control (shScr). shRNA targeting mouse Shp2 or non‐targeting sequence (shShp2, 5′‐TTGAGACCAAGTGCAACAATT‐3′; shScramble, 5′‐CCTAAGGTTAAGTCGCCCTCG‐3′).

### 2.6 Stable Shp2‐KD cell line

Lentiviruses were generated in HEK293T cells as the shRNA pLKO.1‐puro plasmids were co‐transfected with the packaging plasmids pMD2G and pSPAX2. After 48 h of transfection, viral stocks were harvested from the culture medium. The viral supernatant was filtered to remove cells and debris. MLE‐12 cells were infected with the lentivirus above. To select MLE‐12 cells stably expressing shRNA constructs, we treated these cells with 2 μg/ml of puromycin, 48 h after lentivirus infection. After about 4 weeks of selection, monolayers of stably infected pooled clones were harvested for use and cryopreserved. shRNA targeting mouse Shp2 or non‐targeting sequence (shShp2, 5′‐TTGAGACCAAGTGCAACAATT‐3′; shScramble, 5′‐CCTAAGGTTAAGTCGCCCTCG‐3′).

### 2.7 Western blot

Cells were lysed in RIPA lysis buffer containing PMSF (Beyotime) and Protease Inhibitor Cocktail (Roche), then protein concentration was quantified by Bradford assay (Bio‐Rad). Additionally, sEVs were directly lysed in 1× SDS loading buffer.

The samples were boiled and then separated by tris‐glycine SDS–PAGE, electro‐transferred to nitrocellulose membranes (Pall). After being blocked in 5% milk or BSA (w/v), membranes were incubated with indicated primary antibodies, followed by IRDye 680/800 secondary antibodies (LI‐COR). Signals were visualized with Odyssey CLx (LI‐COR) and were quantified by Image studio.

### 2.8 Co‐immunoprecipitation (Co‐IP)

Co‐IP was performed using the SureBeads Protein A/G (Bio‐Rad) according to the manufacturer's instructions. Briefly, cells were lysed in NP‐40 lysis buffer containing PMSF and Protease Inhibitor Cocktail at 4°C for 30 min. Then lysates were cleared by centrifugation at 4°C, 14,000 × *g* for 10 min. Then the supernatants were added with antibody‐conjugated magnetic beads and incubated overnight at 4°C. After that, beads were washed and finally eluted with 1 × SDS loading buffer for western blot analysis.

### 2.9 Fluorescence microscopy

For immunofluorescence staining, tissues or cells were fixed in 4% PFA (pH 7.0). Samples were permeabilized with 0.5% Triton X‐100 for 50 min before blocking with 4% goat serum for 1 h. Samples were incubated with primary antibody at 4°C overnight followed by secondary antibody incubation. Images were collected on an inverted confocal microscope.

The stained cells or tissues were viewed under an inverted Olympus FluoView FV1000 confocal microscope and 40× or 60 × /1.40 NA objective lens, imaged using an Olympus FluoView version 4.2b viewer (Olympus). Images of the cells or tissues were captured, and positive areas were analyzed.

For microscopic analysis of Syntenin‐mCherry inside the lumen of enlarged endosomes, cells were grown on glass coverslips in 24‐well or 12‐well plates prior to transfection. The cells were transfected using Lipo2000 (Invitrogen) according the manual. Then cells were transfected with Syntenin‐mCherry or its mutant and RAB5(Q79L)‐GFP. After 24 h, the cells were inhibited with SHP099 (20 μM). 24 h later, cells were washed once with PBS and fixed for 20 min at room temperature (RT) with 4% paraformaldehyde. After fixation, cells were permeabilized with 0.1% Triton X‐100 in PBS, followed by blocking with PBS‐ 10% foetal serum. Coverslips were mounted with Fluoromount (SouthernBiotech). The intensity of the images was obtained using an inverted Olympus FluoView FV1000 confocal microscope. Syntenin‐mCherry fluorescence signals in the lumen of RAB5(Q79L)‐GFP‐positive endosomes indicate relative ILV numbers.

In the in vitro donor–acceptor sEV transfer system, acceptors in the lower chamber were analyzed by fluorescence microscopy. Acceptors were grown on glass coverslips in 24‐well prior to sEV transfer. For microscopic analysis of PKH67 intensity in acceptors, cells were treated following the above‐mentioned steps. PKH67 fluorescence signals in the acceptors indicate the amount of sEVs transferring from donors.

To analyze Shp2 level in mouse alveolar type II (ATII) epithelial cells, we conducted immunostaining assay. ATII cells isolated from Ctr or Shp2 cKO mice (ATII conditional Shp2 KO) were grown on glass coverslips in 24‐well. Cells were manipulated following the protocol previously described (Bhandary et al., 2013). Cells were incubated with the indicated primary antibodies for 12 h overnight at 4°C, followed by incubation with secondary antibodies for 1 h, diluted in blocking buffer (Alexa Fluor Donkey anti‐Mouse‐488 or Alexa Fluor Donkey anti‐Rabbit‐555). Coverslips were mounted with Fluoromount (SouthernBiotech). The intensity of the images was obtained using an inverted Olympus FluoView FV1000 confocal microscope.

### 2.10 Recombinant protein purification

GST‐tagged fusion proteins (human Shp2 full length, human Shp2 PTP domain, human Src full length, human wildtype Syntenin, human Syntenin Y46F mutant) were purified from BL21 cells. Cells were allowed to grow in constant shaking (220 rpm) at 37°C until log phase, then induced with 0.5 mM IPTG at 16°C for 12 h. Harvested by centrifugation, cells were resuspended in lysis buffer (20 mM Tris‐HCL PH 7.5, 300 mM NaCl, 1% Triton X‐100 and Protease Inhibitor Cocktail). After sonication, cell debris was removed by centrifugation. The supernatant was incubated with glutathione‐sepharose overnight at 4°C. For in vitro phosphorylation and dephosphorylation assays, the bound proteins were eluted with washing buffer containing 20 mM Glutathione or cleaved with HRV 3C protease for fusion tag.

### 2.11 In vitro phosphorylation and dephosphorylation assays

Purified from E. Coli, GST‐tagged wild‐type Syntenin or the Y46F mutant proteins (10 nM) were incubated with recombinant Src (10 nM) in 100 μl reaction buffer (20 mM HEPES, pH 7.5, 150 mM NaCl, 10 mM MgCl_2_ and 10 mM BME), in the presence or absence of 5 mM ATP at 37°C for 30 min. Thereafter, 1 ml of washing buffer PBS‐T (PBS + 0.1% Tween 20) was added along with glutathione sepharose to bind GST‐Syntenin and dilute ATP/MgCl_2_. After 30 min binding at 4°C, sepharose beads were separated from the solution. These beads with bound proteins were washed five times in washing buffer and the final washing was conducted in 100 μl dephosphorylation buffer (60 mM HEPES, pH 7.2, 75 mM NaCl, 75 mM KCl, 1 mM EDTA, 0.05 % Tween 20, 5 mM DTT). Subsequently, phosphorylated Syntenin (10 nM) was incubated with recombinant Shp2 full length (40 nM) or Shp2 PTP domain (10 nM) in dephosphorylation buffer at 37°C for 1 h. The beads‐bound Syntenin were then washed five times with washing buffer and finally eluted with loading buffer for western blot analysis. Phosphorylated Syntenin were detected by anti‐pTyr antibody.

This method was determined according to Prof. J. E. Ladbury's (University of Leeds) guidance and protocol (Ahmed et al., 2013). Prof. J. E. Ladbury generously provided details about the specific ingredients of dephosphorylation buffer. Under his recommendation, we used 1:1 ratio of substrate (10 nM) and kinase (10 nM) for maximum level of phosphorylated proteins. Phosphorylated Syntenin (10 nM) and Shp2 PTP domain (10 nM) were also mixed 1:1 for reaction. Considering the autoinhibitory mechanism of Shp2 activity, we added more full‐length Shp2 (40 nM) for dephosphorylation assay.  

### 2.12 Real‐time PCR

Total RNA was extracted by Trizol and then reverse‐transcribed into cDNA using ReverTraAce qPCR RT kit (Toyobo). Real‐time PCR was conducted using a SYBR Green reagent (CWBIO) on CFX96 Touch Real‐Time PCR Detection System (Bio‐Rad). Primer sequences are listed as following:

Mouse IL1β (F: TGTGAATGCCACCTTTTGACA and R: GGTCAAAGGTTTGGAAGCAG);
Mouse IL6 (F: CTGCAAGAGACTTCCATCCAG and R: AGTGGTATAGACAGGTCTGTTGG);
Mouse TNFα (F: CAGGCGGTGCCTATGTCTC and R: CGATCACCCCGAAGTTCAGTAG).
Gene expression is shown as fold change relative to control, following normalization to housekeeping gene mouse β‐Actin (F: GGCTGTATTCCCCTCCATCG and R: CCAGTTGGTAACAATGCCATGT).

### 2.13 Sample preparation for mass spectrometry analysis

The cells or sEVs were washed with pre‐cold PBS, collected and lysed in ST buffer (300 mM Tris‐HCl pH 7.6, 2% SDS), incubated at 95°C for 5 min. After centrifugation, the cooled supernatant was transferred to a new tube and reduced with DTT. Mixed protein (200 μg) with 8 M UA in the filter unit (10K), and discarded the flow‐through after centrifugation. Alkylated the protein with IAA and incubated 45 min in the dark. Discarded the flow‐through. Then the sample was dissolved and centrifugated in UA and ABC (50 mM) in sequence. Changed a new collection tube, added 4 μg trypsin and incubated at 37°C for 16 h. Then the flow‐through was collected to a new tube and added 50 mM ABC. After that, collected the flow‐through to the above tube and dried the sample with SpeedVac. At last, the sample was resuspended with 0.1% formic acid for MS analysis.

### 2.14 Mass spectrometry analysis

The peptide samples were analyzed on Thermo Fisher LTQ Obitrap ETD mass spectrometry, briefly, loaded sample onto an HPLC chromatography system named Thermo Fisher Easy‐nLC 1000 equipped with a C18 column (1.8 mm, 0.15 × 1,00 mm). Solvent A contained 0.1% formic acid and solvent B contained 100% acetonitrile. The elution gradient was from 4% to 18% in 182 min, 18% to 90% in 13 min solvent B at a flow rate of 300 nL/min. Mass spectrometry analysis were carried out carried out at the AIMSMASS Co., Ltd. (Shanghai, China) in the positive‐ion mode with an automated data‐dependent MS/MS analysis with full scans (350‐1600 m/z) acquired using FTMS at a mass resolution of 30,000 and the 10 most intense precursor ions were selected for MS/MS. The MS/MS was acquired using higher‐energy collision dissociation at 35% collision energy at a mass resolution of 15,000.

### 2.15 Bioinformatic analysis

Raw MS files were analyzed by MaxQuant (version 1.5.2.8), the parameter used for data analysis included trypsin as the protease with a maximum of two missed cleavages allowed. The mass tolerance for precursor ions and fragment ions was set to 20 ppm and 4.5 ppm, respectively. The search included variable modifications of methionine oxidation and deamidation, and fixed modification of carbamidomethyl cysteine. Minimal peptide length was set to six amino acids and a maximum of two miscleavages was allowed. The false discovery rate (FDR) was set to 0.01 for peptide and protein identifications.

The Uniprot accession number of proteins was uploaded to the Database for Annotation, Visualization and Integrated Discovery (DAVID) bioinformatics resources (v6.8). The cellular component, molecular function and biological process for the proteins were extracted and plotted with Origin. We conducted GO term enrichment analysis and the data were plotted with Origin.

### 2.16 TEM and Cryo‐EM

For transmission electron microscopy (TEM) analysis of sEVs, samples were processed by negative staining. A 200‐mesh carbon film was hydrophilized by glow discharge instrument at 15 mA, 25 s. A 2% uranium acetate was filtered by 0.2 μm membrane. A 3 μL of sEV solution was pipetted onto the carbon film and incubated for 1 min. The carbon film was wash twice with double distilled water. A 5 μl of 2% uranium acetate was pipetted onto the carbon film and incubated for 1 min. Then we discarded the staining solution and let the carbon film dry naturally. Images were acquired using a TEM instrument (Tecnai G2 Spirit, Thermo FEI) at 120 kV.

For MVB analysis of ATII cells, the lung tissues were placed in a droplet of 2.5% glutaraldehyde in PBS buffer and fixed overnight at 4°C. The samples were rinsed three times in PBS for 10 min each and then fixed in 1% osmium tetroxide for 60 min at room temperature. The samples were rinsed three times in PBS again. Then the samples were stained in 2% uranium acetate for 30 min. After that, the samples were dehydrated through a graded series of ethanol concentration eventually reaching 100%. Next, the samples were infiltrated in a 1:1 solution of epon: acetone for 2 h. Then, the samples were infiltrated in a 3:1 solution of epon: acetone overnight. The next day, they were placed in fresh epon for several hours and then embedded in epon overnight at 60°C. Thin sections were cut on an ultramicrotome (UC7, Leica), collected on grids and examined in a TEM instrument (Tecnai G2 Spirit, Thermo FEI) at 120 kV.

For Cryogenic Electron Microscopy (Cryo‐EM), sEVs were vitrified by plunge freezing (Leica EM GP). In brief, sEVs were loaded onto holey carbon grids (200 mesh, Quantifoil), followed by plunge‐freezing at 95% humidity and 25°C. The state of vitreous water in the frozen samples was verified by electron diffraction.

A 5 μl drop of sEVs in PBS was applied to the grids (Quantifoil 1.2/1.3). Grids were then plunge‐frozen in liquid‐nitrogen‐cooled ethane with a freeze unit (Thermo Fisher, Vitrobot). Specimens were transferred under liquid N_2_ temperatures into a 200‐kV FEG TEM (Thermo Fisher, Talos F200C) and observed with a CCD camera (Thermo Fisher, Ceta). The estimated total electron dose for the EM imaging is 70 e‐/Ǻ2.

### 2.17 In vitro donor–acceptor sEV transfer system

In order to characterize sEV uptake and internalization in acceptor cells, donor cells were labelled with PKH67 (Sigma). Donor cells were seeded into the upper chamber of the 0.4 μm transwell insert. Then acceptor cells were grown on the bottom of the lower chamber. After 24 h co‐culture with PBS or LPS (100 ng/ml), the fluorescence signals in lower chamber were detected using fluorescence microscope (FluoView FV1000, Olympus) or flow cytometry (NovoCyte, ACEA). PKH67 fluorescence signals in the acceptors indicate the amount of sEVs transferring from donors.

### 2.18 PKH67 signal detection by flow cytometry

In the donor–acceptor sEV transfer system, the acceptor cells were digested to obtain the single‐cell suspension. Then the suspension was centrifugated and washed twice with PBS for flow cytometry analysis. PKH67 fluorescence signals in the acceptors were determined by 488 nm excitation in NovoCyte Flow Cytometer (ACEA). Data were analyzed with FlowJo software 7.6.

### 2.19 Flow cytometry analysis of sEVs by latex beads

This protocol was performed at 4°C. sEVs were incubated with 5 μl aldehyde/sulfate latex beads (Invitrogen) with 20 μl final volume in PBS. The mixture was incubated 30 min and resuspended every 5 min by gentle pipetting. The sample was blocked by incubation with 400 μl of [PBS/5% BSA] solution for 30 min. After centrifugation (1000 ×*g* for 10 min at 4°C), the pellet was resuspended in 100 μl [PBS/5%BSA] solution and incubated with antibody diluted at 1/200 for 30 min. For detection of sEVs, anti‐CD9 antibody was utilized. A negative control was processed by the same protocol but skipping this staining step. The bead‐bound sEVs were then washed in PBS followed by centrifugation. The pellets were resuspended in 300 μl PBS and analyzed by NovoCyte Flow Cytometer.

### 2.20 Nanoscale flow cytometry

sEVs purified from cell supernatants were diluted in PBS in a final volume of 100 μl. Anti‐mouse CD63 APC‐conjugated antibody (eBioscience), anti‐mouse CD9 FITC‐conjugated (eBioscience) and anti‐mouse CD81 PE‐conjugated (Biolegend) antibody were added to the sEV solution at optimal pre‐determined concentrations and incubated for 30 min at room temperature. Then the samples were analyzed by the CytoFLEX flow cytometer (Beckman Coulter). The cytometer was calibrated using a mixture of non‐fluorescent silica beads and fluorescent (green) latex beads with sizes ranging from 50 nm to 500 nm. By this calibration step, we determined the sensitivity and resolution of the flow cytometer (fluorescent latex beads) and the size of extracellular vesicles (silica beads).

### 2.21 Cell viability assay (CCK‐8 assay)

The cell viability was detected by Cell Counting Kit‐8 (K1018, ApexBio) according to manufacturer's instructions. DMSO served as the solvent control. After treatment, 10 μl of CCK‐8 solution was added to each well. The samples were incubated at 37°C for 1 h. Then the OD value for each well was detected at 450 nm on a microplate reader (M5, Molecular Devices). The assay was repeated three times.

### 2.22 Mice

SPC‐CreERT2 mice (The Jackson Laboratories) were crossed with Shp2^flox/flox^ mice (C57BL/6 background) to generate mice with conditional Shp2 knockout (KO) in ATII cells. LysM^Cre/+^ mice were mated with Shp2^flox/flox^ mice to generate conditional Shp2 KO mice. Shp2^flox/flox^ mice were used as controls, SPC^CreERT2/+^:Shp2^flox/flox^ and LysM^Cre/+^: Shp2^flox/flox^ were used as mutant mice (designated as Ctr and cKO in this study, respectively) and were selected to be used in the experiments. To confirm the excision of Shp2, we used a forward (5′‐CAGTTGCAACTTTCTTACCTC‐3′) primer and a reverse (5′‐GCAGGAGACTGCAGCTCAGTGATG‐3′) primer within introns 3 and 4, respectively.

CreERT2 recombinase activity in these transgenic mice was induced by tamoxifen (Sigma). Tamoxifen was dissolved in a corn oil/ethanol mixture at 10 mg/ml. Each 6–8 week old mouse was injected intraperitoneally with 100 μl of tamoxifen per day for 5 consecutive days. One week after the last injection, age‐matched Shp2‐conditional KO and littermate control mice were used for experiments. For LysM^Cre/+^: Shp2^flox/flox^ and Shp2^flox/flox^ mice, bone marrow–derived macrophages (BMDMs) were isolated and differentiated as previously described (Xu et al., 2017).

Mice were kept under specific pathogen–free condition. All animal protocols were approved by the Animal Care and Use Committee of the Zhejiang University School of Medicine.

### 2.23 Isolation of mouse alveolar macrophages

Alveolar macrophages (AMs) were harvested by bronchoalveolar lavage (BAL). Here, we described an adapted protocol as following (Busch et al., 2019). Mice were euthanized. Lungs were lavaged with 0.5 ml of warm BAL buffer (2 mM EDTA and 0.5% FBS in PBS) for nine times. Then the collected BAL fluid (BALF) was cooled on ice and filtered through a 70 μm cell strainer into the 15‐ml tube with 1.5 ml complete medium (RPMI 1640 supplemented with 10% FBS with penicillin and streptomycin). Filtered BALF was centrifuged at 300 × *g*, 5 min at 4°C. Remove supernatant. A 1 ml haemolysis buffer was added for 2 min incubation at room temperature to lyse residual erythrocytes. Complete medium was added to stop lysis and cells were collected by centrifugation as before. Remove supernatant. Resuspend the pelleted cells and plate with complete medium. Then we collected the adherent cells after 2 h to 4 h of cultivation. For PLD2i (PLD2 inhibition) administration, mice were intraperitoneally injected CAY10594 (2 mg/kg). After 4 h, mice were sacrificed for AM isolation.

### 2.24 Isolation of mouse alveolar type II (ATII) epithelial cells

Mouse ATII cells were isolated according to the method described previously (Bhandary et al., 2013). ATII cell purity was confirmed via lithium carbonate staining (Dobbs, 1990).

### 2.25 Histopathology

Left lung tissues from the experimental mice were fixed in formalin and embedded in paraffin. For the histological analysis, the paraffin‐containing samples were sectioned and stained with haematoxylin and eosin (HE) according to the standard procedure. The HE‐stained slides were examined by optical microscopy (Eclipse Ci‐S; Nikon).

### 2.26 Data analysis and statistics

Data analyzes were performed with a one‐way ANOVA or a two‐tailed Student's *t*‐test using GraphPad Prisantibodym version 5.0 (GraphPad, La Jolla, CA, USA). The number of experimental repeats and larvae are indicated in the relevant figure legends. A *P*‐value < 0.05 was considered significant and is indicated in the figures as *(*P* < 0.05), **(*P* < 0.01) or ***(*P* < 0.001).

## 3 RESULTS

### 3.1 Phosphatase Shp2 controls secretion of sEVs in vitro

**FIGURE 1:**
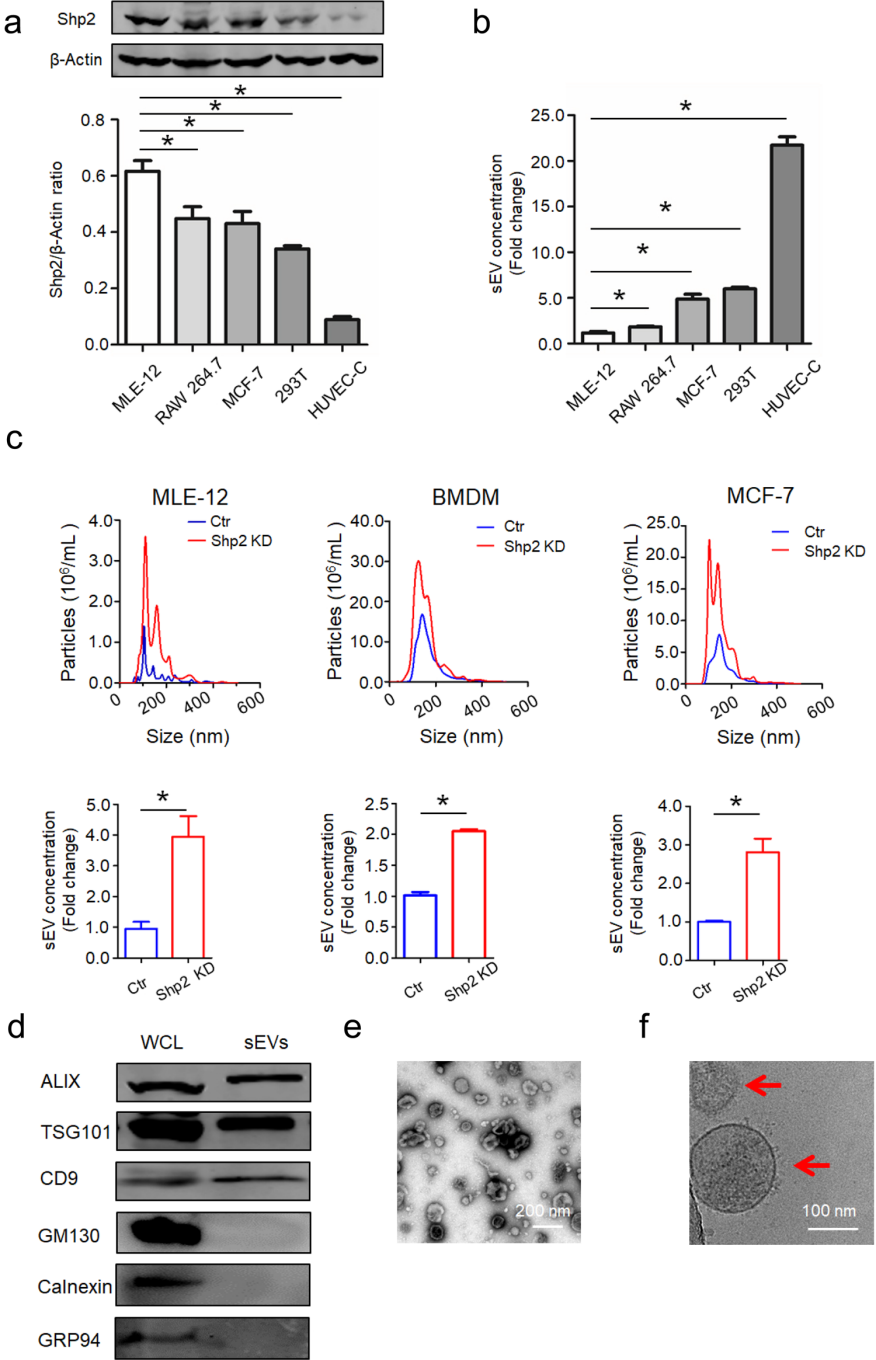
Phosphatase Shp2 regulates secretion of small extracellular vesicles in vitro. [(a) Relative protein level of Shp2 and (b) sEV concentration in different cell lines. sEV concentration represents the quantity of sEVs released by same number of cells. Fold change is compared to MLE‐12 cells. (c) NanoSight quantification of sEV numbers in control and Shp2 KD cells. sEV concentration represents the quantity of sEVs released by same number of cells. Fold change is compared to control. (d) Western blot analysis of sEVs purified from cell culture supernatants from MLE‐12 cells. Whole cell lysates (WCL) and sEVs were blotted for EV‐markers ALIX, TSG101, CD9, and negative control GM130, Calnexin and GRP94.(e)Negatively stained TEM image and (f) Cryo‐EM image (indicated by arrowheads) show purified sEVs from MLE‐12 cells. Data from three independent experiments are shown. *P < 0.05, **P < 0.01, and ***P < 0.001]

It is reported that many kinases promote sEV production by phosphorylating downstream signal molecules (Dautova et al., 2018; Hirsova et al., 2016; Imjeti et al., 2017; Lee et al., 2018; Wei et al., 2017; Zhang et al., 2018). In order to preliminarily investigate the corresponding role of phosphatases in sEV biogenesis, we treated cells with available inhibitors for several widely expressed phosphatases. The working concentration of different inhibitors was determined by cell viability measured by CCK‐8 assay (Figure S1). Administrated phosphatase inhibitors promoted sEV release in different degrees (Figure S2A and B). We found inhibition of tyrosine phosphatase Shp2 increased sEV secretion. Reportedly, Shp2 counteracts the Src‐mediated effects on VIF tyrosine phosphorylation and organization (Yang et al., 2019). Furthermore, as the regulatory binding partner of phosphorylated Y720, Shp2 mediates PDGFRα enrichment in EVs and regulates HSC‐derived EV release (Gao et al., 2020; Kostallari et al., 2018). Therefore, we further investigated the dephosphorylating function of Shp2 in sEV formation. First, we detected its expression level in different cell lines (Figure 1a). In Figure 1b, the corresponding sEV concentration was analyzed by a Nanosight NS 500 system. Interestingly, the sEV number was negatively correlated to the Shp2 level (Figure 1b). To validate the inhibitory function of Shp2 in sEV biogenesis, we detected the sEV concentration of MLE‐12, BMDM (Bone marrow derived macrophages) and MCF‐7 cells by Shp2 KD (Figure S3A). The results showed that Shp2 KD led to upregulated sEV secretion (Figure 1c). Moreover, we also detected the increased sEV concentration of Shp2 inhibited (BMDM, MCF‐7) and Shp2 KO (BMDM) cells (Figure S3B). In addition, NTA analysis confirmed that the sizes of released sEVs were mainly around 100 nm. sEVs were isolated from cell culture supernatant by ultracentrifugation and characterized by transmission electron microscopy (TEM), Cyro‐EM and western blot (WB) (Figure 1c‐f).

**FIGURE 2:**
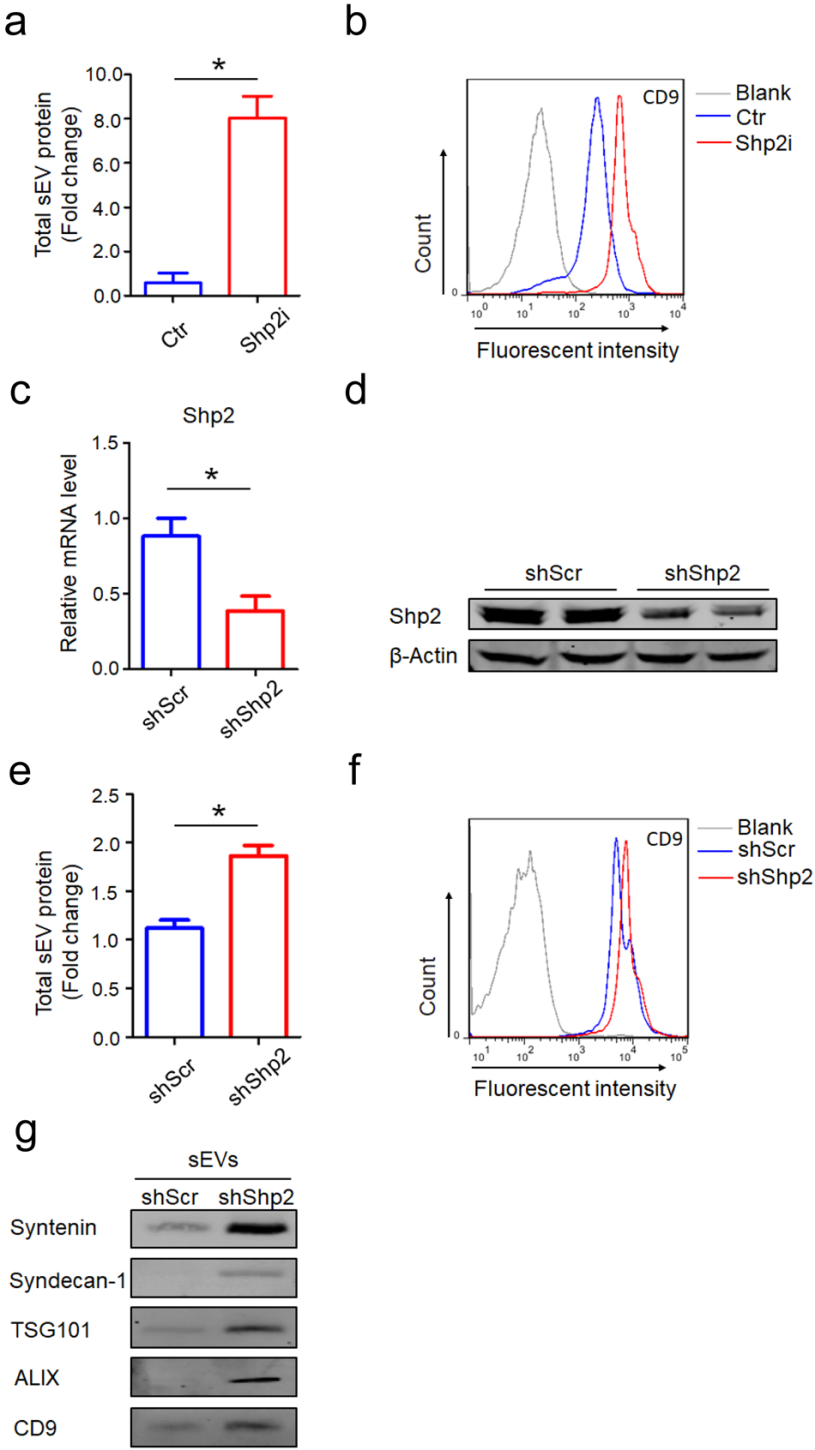
Pharmacologic inhibition and shRNA‐mediated down‐regulation of Shp2 increases sEV secretion of epithelial cells. [(a) Quantification of protein levels in the sEVs obtained from Ctr (DMSO) and Shp2i (PHPS1 20 μM, Shp2i is short for Shp2 inhibition) treated cells (MLE‐12 cells). Fold change is compared to Ctr. (b) After being absorbed on latex beads, sEVs obtained from Ctr (DMSO) and Shp2i (PHPS1 20 μM) treated cells (MLE‐12 cells) were analyzed by flow cytometry with the CD9 antibody.(c) Efficiency of Shp2 depletion in Shp2 KD stable epithelial cell lines (MLE‐12 cells). (d) Western blot analysis of Shp2 in Shp2 KD stable epithelial cell lines (MLE‐12 cells). (e) Quantification of protein levels in the sEVs obtained from shScr and shShp2 stable epithelial cell lines (MLE‐12 cells). Fold change is compared to shScr. (f) After being absorbed on latex beads, sEVs obtained from shScr and shShp2 stable epithelial cell lines (MLE‐12 cells) were analyzed by flow cytometry with the CD9 antibody. (g) Western blot analysis of sEVs purified from cell culture supernatants from equal numbers of shScr and shShp2 stable epithelial cell lines (MLE‐12 cells). sEVs were blotted for Syntenin, Syndecan‐1, TSG101, ALIX and CD9. Data from three independent experiments are shown. *P < 0.05, **P < 0.01, and ***P < 0.001]

Measured by total sEV protein or surface antigen CD9, sEV number was increased by Shp2 inhibition in MLE‐12 cells (Figure 2a and b). To further investigate the function of Shp2 in regulating sEVs in vitro, we constructed Shp2‐KD stable MLE‐12 cells (Figure 2c and d). Moreover, sEV number was increased by Shp2 stable KD in MLE‐12 cells, indicated by total sEV protein or surface antigen CD9 (Figure 2e and f). Shp2 stable KD in MLE‐12 cells resulted in a significant increase of Syntenin, Syndecan‐1, TSG101, ALIX and CD9 in sEVs, suggesting the enhanced sEV secretion (Figure 2g). These results supported the concept that Shp2 inhibited sEV biogenesis, therefore we further investigated the mechanism Shp2 is involved in.

### 3.2 Shp2 regulates ILV formation in MVBs

**FIGURE 3:**
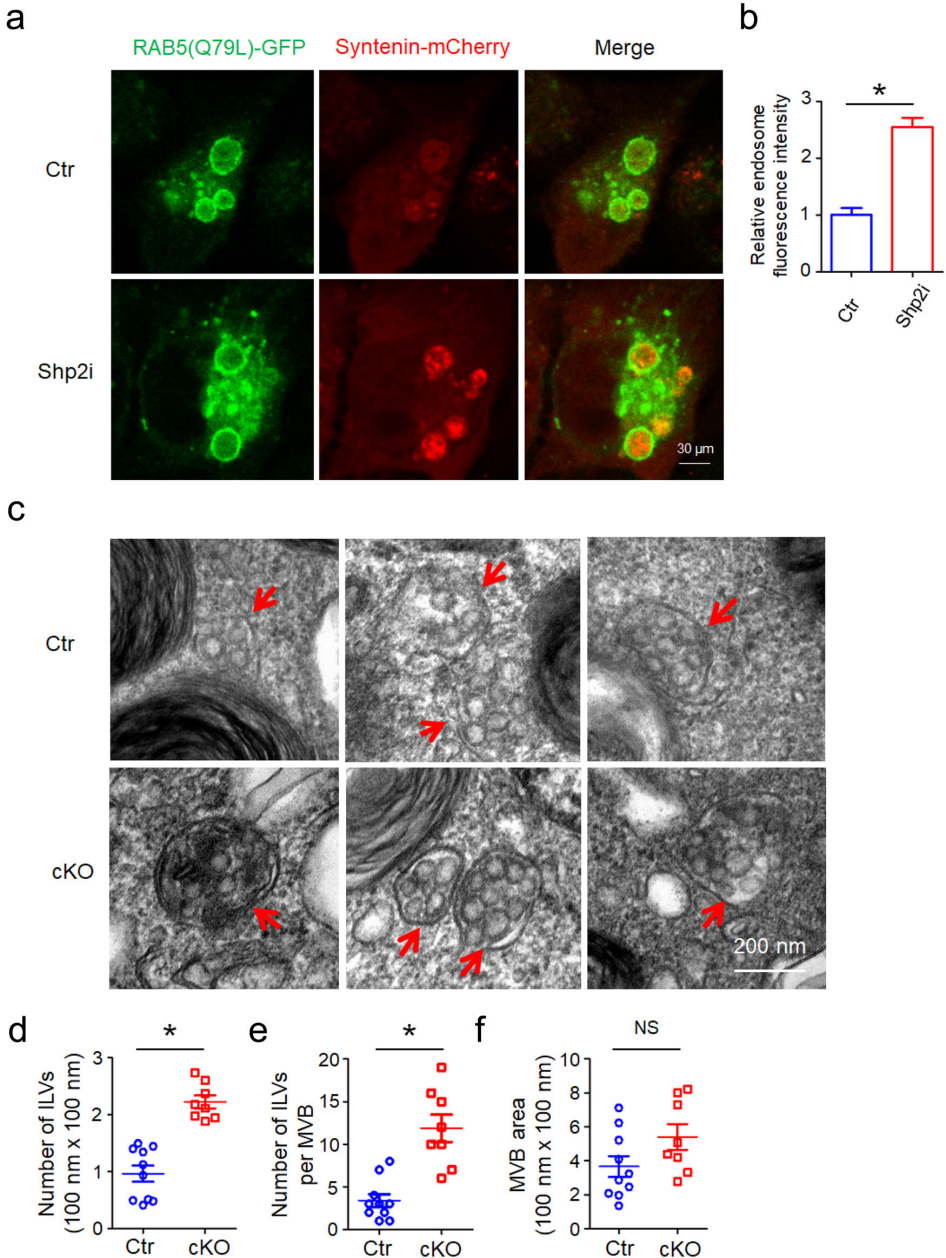
Increases in number of intracellular vesicles (ILVs) by Shp2 inhibition or depletion. [(a) Confocal micrographs show the accumulation of Syntenin‐mCherry inside the lumen of enlarged endosomes outlined by RAB5(Q79L)‐GFP in Ctr (DMSO) and Shp2i (SHP099 20 μM) treated cells (MCF‐7). (b) Relative fluorescence intensity of Syntenin‐mCherry in RAB5(Q79L)‐GFP endosomes indicates relative ILV numbers. Each quantification was performed considering at least 30–50 RAB5(Q79L) endosomes. (c) Representative TEM images of ATII cells in Ctr and cKO (ATII conditional Shp2 KO) mice. Red arrows indicate MVBs containing typical intraluminal vesicles (ILVs). (d) The ILV density in MVBs. (e) The number of ILVs per MVB. (f) Quantitative analysis of MVB area. The ILV density, number of ILVs per MVB and MVB area in 8–10 profiles of different ATII cells were counted and only MVBs containing typical ILVs were counted. Data from three independent experiments are shown. *P < 0.05, **P < 0.01, and ***P < 0.001. NS, non significant]

ILVs are generated in MVBs and secreted to extracellular environment as sEVs (Babst, 2011). To further study the role of Shp2 in regulating ILV biogenesis in MVBs, we utilized the RAB5(Q79L) mutant. The GTPase‐deficient Rab5 mutant, Rab5(Q79L), stimulates endosomal fusion, causing the formation of enlarged multivesicular bodies (Imjeti et al., 2017). RAB5(Q79L)‐GFP was expressed along with Syntenin‐mCherry, which indicated ILVs. Therefore, we analyzed the effect of Shp2 inhibition on the accumulation of Syntenin‐mCherry inside endosomes outlined by RAB5(Q79L)‐GFP. In Shp2‐inhibited cells, confocal microscopy revealed increased accumulation of Syntenin‐mCherry inside the lumen of RAB5(Q79L)‐GFP endosomes (Figure 3a‐b).

We investigated the number of ILVs in MVBs by electron microscopy. Consistent with in vitro results, the density and number of ILVs per MVB significantly increased by Shp2 depletion in alveolar type II (ATII) epithelial cells (Figure S4), indicating that the absence of Shp2 results in the enhanced accumulation of ILVs in MVBs (Figure 3c‐e). Meanwhile, the size of MVBs did not show any significant changes, suggesting that Shp2 only affects ILV numbers rather than MVB size (Figure 3f). In summary, these results suggest that Shp2 pharmacologic inhibition and depletion‐mediated mounting numbers of ILVs in MVBs led to upregulated sEV release.

### 3.3 Comparative analysis of sEVs from control and Shp2 stable KD epithelial cells

Subtypes and components of sEVs determine their function in patho/physiological conditions (Kowal et al., 2016). To make comprehensive comparison, the protein composition of sEVs from control and Shp2 stable KD epithelial cells (MLE‐12 cells) were studied via mass spectrometry (MS). To compare functional contents of sEVs, we conducted GO analysis on the list of proteins present in sEV samples. After GO analysis, genes were assigned to GO terms including molecular functions, cellular components or biological processes. We found that sEV proteins were both enriched in Metabolic process, Localization, Catalytic activity, Protein‐containing complex, Transporter activity, Membrane, Extracellular region and Cellular component organization or biogenesis (Figure S5). Furthermore, GO analysis detected no significant difference in the proportion and constitution of Go terms between these two groups (Figure S5). This result suggests that loss of phosphatase Shp2 does not significantly change the sEV protein components in MLE‐12 cells. Moreover, the sEVs were analyzed using nanoscale flow cytometry (CytoFlex). As a result, Shp2 stable KD did not significantly alter the sEV subpopulations of MLE‐12 cells, indicated by CD9, CD63 and CD81 (Figure S6). We concluded that Shp2 worked on sEVs without significantly changing its subpopulations or composition.

### 3.4 Syntenin Tyrosine 46 is the regulatory phosphorylation site dephosphorylated by Shp2

To investigate the molecular mechanisms of Shp2 in regulating sEV biogenesis, we performed MS‐based quantitative proteomics to analyze the differentially expressed proteins in Shp2 stable KD MLE‐12 cells compared with control (Figure S7). We analyzed reported proteins involved in sEV formation (Hessvik & Llorente, 2018; Kowal et al., 2014; Song et al., 2019). As shown, the analyzed proteins were not changed significantly (*P* < 0.05) or greatly (Fold change > 2) (Figure S8). This result was verified by detecting the expression level of target proteins (Figure S9A).

**FIGURE 4:**
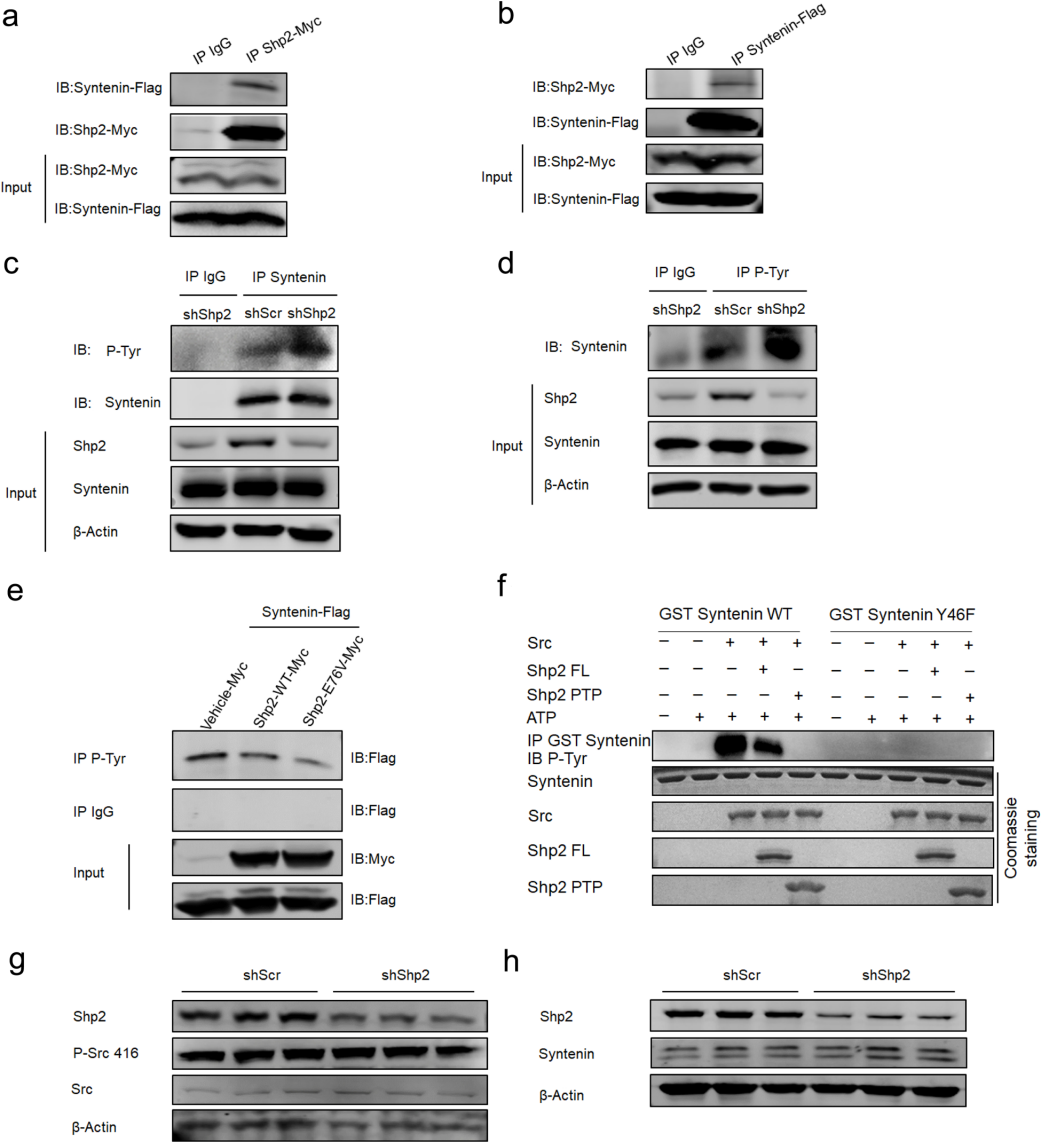
Shp2 dephosphorylates tyrosine 46 of Syntenin. [(a) 293T cells were cotransfected with Shp2‐Myc and Syntenin‐Flag. The cells were lysed and immunoprecipitated (IP) using anti‐Myc antibody or IgG. The interaction between Shp2‐Myc and Syntenin‐Flag was detected by western blot with the anti‐Flag antibody. (b) 293T cells were cotransfected with Shp2‐Myc and Syntenin‐Flag. The cells were lysed and immunoprecipitated (IP) using anti‐Flag antibody or IgG. The interaction between Shp2‐Myc and Syntenin‐Flag was detected by western blot with the anti‐Myc antibody. (c)shScr and shShp2 stable epithelial cells (MLE‐12 cells) were lysed and immunoprecipitated (IP) using anti‐Syntenin antibody or IgG. The tyrosine phosphorylation level of Syntenin was detected by western blot with the anti‐P‐Tyr antibody. (d) shScr and shShp2 stable epithelial cells (MLE‐12 cells) were lysed and immunoprecipitated (IP) using anti‐ P‐Tyr antibody or IgG. The tyrosine phosphorylation level of Syntenin was detected by western blot with the anti‐Syntenin antibody. (e) 293T cells were cotransfected with Syntenin‐Flag and Vehicle‐Myc, Shp2‐WT‐Myc Shp2‐E76V‐Myc (constitutively active form) respectively. The cells were lysed and immunoprecipitated (IP) using anti‐P‐Tyr antibody or IgG. The tyrosine phosphorylation level of Syntenin was detected by western blot with the anti‐Flag antibody. (f) In vitro dephosphorylation of the Src‐phosphorylated GST‐Syntenin fusion protein by recombinant Shp2 FL (Full‐length) and Shp2 PTP domain in the presence or absence of ATP, and revealed by Western blot, using anti‐ P‐Tyr antibody. (g) Western blot analysis of P‐Src 416 level in shScr and shShp2 MLE‐12 cells. (h) Western blot analysis of Syntenin level in shScr and shShp2 MLE‐12 cells. Data from three independent experiments are shown. *P < 0.05, **P < 0.01, and ***P < 0.001]

To find out Shp2‐interacting proteins involved in sEV biogenesis, we performed Co‐immunoprecipitation (Co‐IP) assays. We found the interaction between Shp2 and Syntenin (Figure 4a‐b) rather than other proteins (Figure S9b‐e). As reported, phosphorylated proteins have been demonstrated to play a pivotal role in regulating the sEVs (Carnino et al., 2020). It is reported that the increase in the tyrosine phosphorylation of Syntenin results in enhanced sEV formation (Imjeti et al., 2017), so we detected the relative function of tyrosine phosphatase Shp2. Co‐IP assay showed that Shp2 stable KD led to increased tyrosine phosphorylation of Syntenin in MLE‐12 cells (Figure 4c‐d). Additionally, overexpression of wildtype or constitutively activated Shp2 mutant (Shp2 E76V) decreased the tyrosine phosphorylation level of Syntenin (Figure 4e). Syntenin controls sEV formation via tyrosine 46 phosphorylation in a Src‐dependent manner (Imjeti et al., 2017). Moreover, we established that recombinant active Shp2 directly dephosphorylated recombinant GST‐syntenin which was beforehand phosphorylated by Src (Figure 4f). We also observed that introduction of a Y46F phosphodeficient mutation of Syntenin blocked the above effects (Figure 4f). Furthermore, Shp2 stable KD did not affect Src activation (P‐416) and Syntenin expression level (Figure 4g‐h). These results suggested that Shp2 directly dephosphorylates tyrosine phosphorylation of Syntenin.

### 3.5 Shp2‐regulated sEV formation is dependent on Syntenin

**FIGURE 5:**
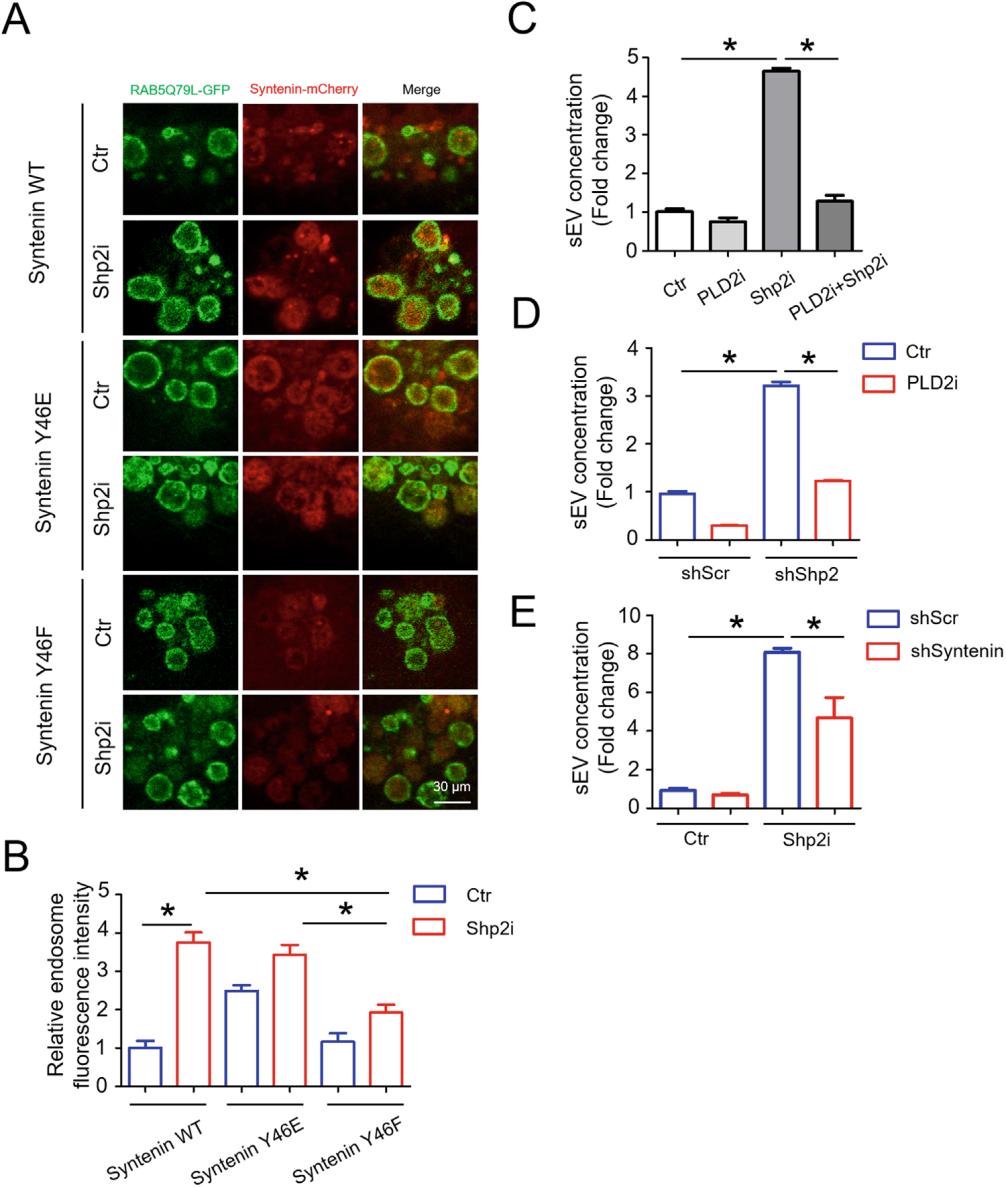
Tyrosine phosphorylation of Syntenin mediates the regulation of Shp2 on sEV formation. [(a) Confocal micrographs of MCF‐7 cells that were transfected with Syntenin‐mCherry WT, Syntenin‐mCherry Y46E and Syntenin‐mCherry Y46F along with the expression vectors encoding RAB5(Q79L)‐GFP for 24 h. Then these cells were treated with Ctr (DMSO) and Shp2i (SHP099 20 μM) for another 24 h. (b) Relative fluorescence intensity of Syntenin‐mCherry in RAB5(Q79L)‐GFP endosomes indicates relative ILV numbers, in the different conditions. Each quantification was performed considering at least 30–50 RAB5(Q79L) endosomes. (c) Epithelial cells (MLE‐12 cells) were treated with Ctr (DMSO), PLD2i (CAY10594, 5 μM), Shp2i (SHP099, 10 μM) or Shp2i + PLD2i for 24 h. sEVs in the culture supernatants were isolated for Nanosight analysis. (d) shScr and shShp2 stable epithelial cells (MLE‐12 cells) were treated with either Ctr (DMSO) or PLD2i (CAY10594, 5 μM) for 24 h. sEVs in the culture supernatants were isolated for Nanosight analysis. (e) Epithelial cells (MLE‐12 cells) were treated with either Syntenin siRNA (siSyntenin) or Scramble siRNA (siScr) for 48 h. Then cells were cultured with fresh medium (sEV‐depleted, with Ctr (DMSO) or Shpi (SHP099, 10 μM)) for another 24 h. sEVs in the culture supernatants were isolated for Nanosight analysis. sEV concentration represents the number of sEVs released by same quantity of cells. Fold change is compared to control. Data from three independent experiments are shown. *P < 0.05, **P < 0.01, and ***P < 0.001]

We thus tested the function of Shp2 in sEV formation by dephosphorylating Syntenin Y46. To assess the role of Syntenin dephosphorylation by Shp2 in sEV biogenesis, we also performed sEV formation experiments in the presence of Shp2 inhibitor. Shp2 inhibition increased Syntenin‐mCherry sEV budding when Syntenin was wildtype but not when Syntenin was phosphodeficient (Y46F) (Figure 5a‐b). As reported, Syntenin Y46F phosphodeficient mutation reduces sEV formation, whereas the phosphomimetic Y46E mutation promotes this process (Imjeti et al., 2017). This suggests that the dephosphorylation of Syntenin by Shp2 inhibits sEV budding. In addition, depleting Syntenin (Figure S9F) and inhibiting its downstream effector PLD2 (Ghossoub et al., 2014) both blocked Shp2 stable KD induced sEV increase (Figure 5c‐e). Taken together, these data indicate that Shp2 controls sEV biogenesis by directly dephosphorylating Syntenin at tyrosine 46.

### 3.6 Enhanced epithelial‐macrophage sEV transfer by epithelial Shp2 stable KD

**FIGURE 6:**
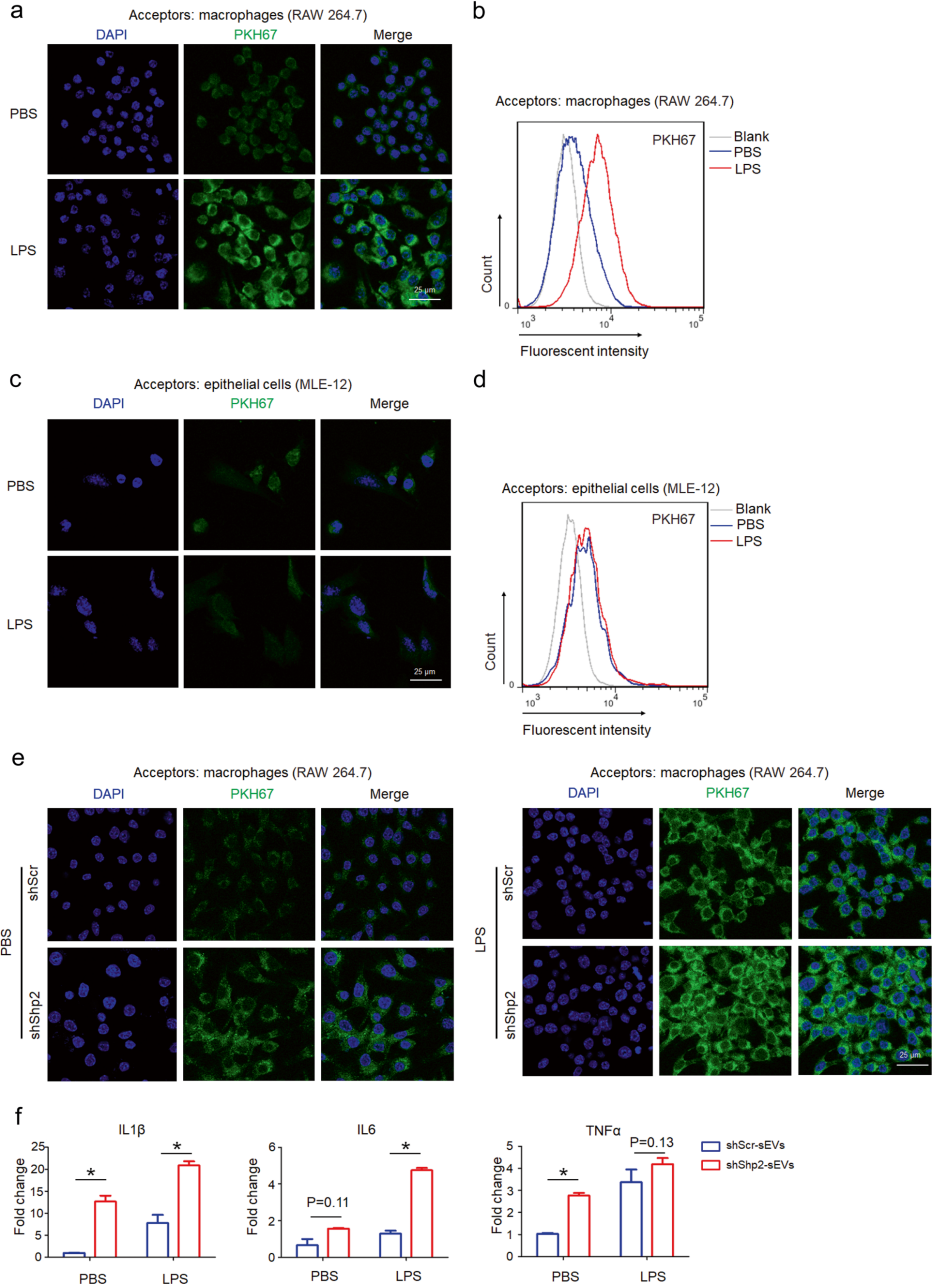
Epithelial Shp2 stable KD promotes sEVs transferring from epithelial cells to macrophages and facilitates macrophage activation in vitro. [(a) Confocal images show uptake of epithelial sEVs by macrophages. PKH 67‐labeled epithelial cells (MLE‐12 cells) were seeded into the upper chamber of the transwell insert. Macrophages (RAW 264.7) were seeded in the lower chamber. After 24 h co‐culture with PBS or LPS (100 ng/ml), macrophages were analyzed by confocal microscopy. (b) Flow cytometry analysis of macrophages in the lower chamber. (c) Confocal images show uptake of macrophage sEVs by epithelial cells. PKH 67‐labeled Macrophages (RAW 264.7) were seeded into the upper chamber of the transwell insert. Epithelial cells (MLE‐12 cells) were seeded in the lower chamber. After 24 h co‐culture with PBS or LPS (100 ng/ml), epithelial cells were analyzed by confocal microscopy. (d) Flow cytometry analysis of epithelial cells in the lower chamber. (e) Confocal images show uptake of epithelial sEVs by macrophages. PKH 67‐labeled shScr and shShp2 stable epithelial cells (MLE‐12 cells) were seeded into the upper chamber of the transwell insert. Macrophages (RAW 264.7) were seeded in the lower chamber. After 24 h co‐culture with PBS or LPS (100 ng/ml), macrophages were analyzed by confocal microscopy. (f) Macrophages (RAW 264.7) were treated with sEVs derived from shScr and shShp2 stable epithelial cell lines (MLE‐12 cells). After 4 h, cells were stimulated with PBS or LPS (100 ng/ml) for 2 h. The mRNA levels of inflammatory cytokine IL1β, IL6 and TNFα were determined by qPCR. Fold change is compared to shScr‐sEVs in PBS. Data from three independent experiments are shown. *P < 0.05, **P < 0.01, and ***P < 0.001]

In alveolar microenvironment, EV‐mediated intercellular communication between lung epithelial cells and alveolar macrophages plays an important role in the pathogenesis of pulmonary diseases (Lee et al., 2018). Lung epithelial cell‐derived extracellular vesicles activate macrophage‐mediated inflammatory responses in acute lung injury (Moon et al., 2015). In order to investigate the role of Shp2 in sEV‐mediated epithelial‐macrophage crosstalk, we established an indirect co‐culture system for in vitro donor–acceptor sEV transfer (Figure S10). The fluorescence intensity of acceptors reflected the amount of PKH67‐stained sEVs transferring from donors. In the LPS‐induced inflammatory microenvironment, sEV transfer tendency was from epithelial cells (MLE‐12 cells) to macrophages (RAW 264.7) (Figure 6a‐b) rather than a reverse pattern (Figure 6c‐d). To study whether Shp2 regulates sEV‐mediated epithelial‐macrophage crosstalk, we seeded Shp2 stable KD MLE‐12 cells into the upper chamber of the transwell insert. The results showed that epithelial Shp2 stable KD enhanced epithelial‐macrophage sEV transfer both in the resting or LPS‐induced inflammatory microenvironment (Figure 6e).

### 3.7 Epithelial Shp2 stable KD induces sEV mediated macrophage activation

To assess the function of Shp2 in epithelial sEV activity, sEVs isolated from MLE‐12 cells (shScr and shShp2) were added into the culture medium of RAW 264.7. sEVs derived from shShp2 stable MLE‐12 cells resulted in upregulated IL1β, IL6 and TNFα expression in acceptor RAW 264.7 (Figure 6f).

**FIGURE 7:**
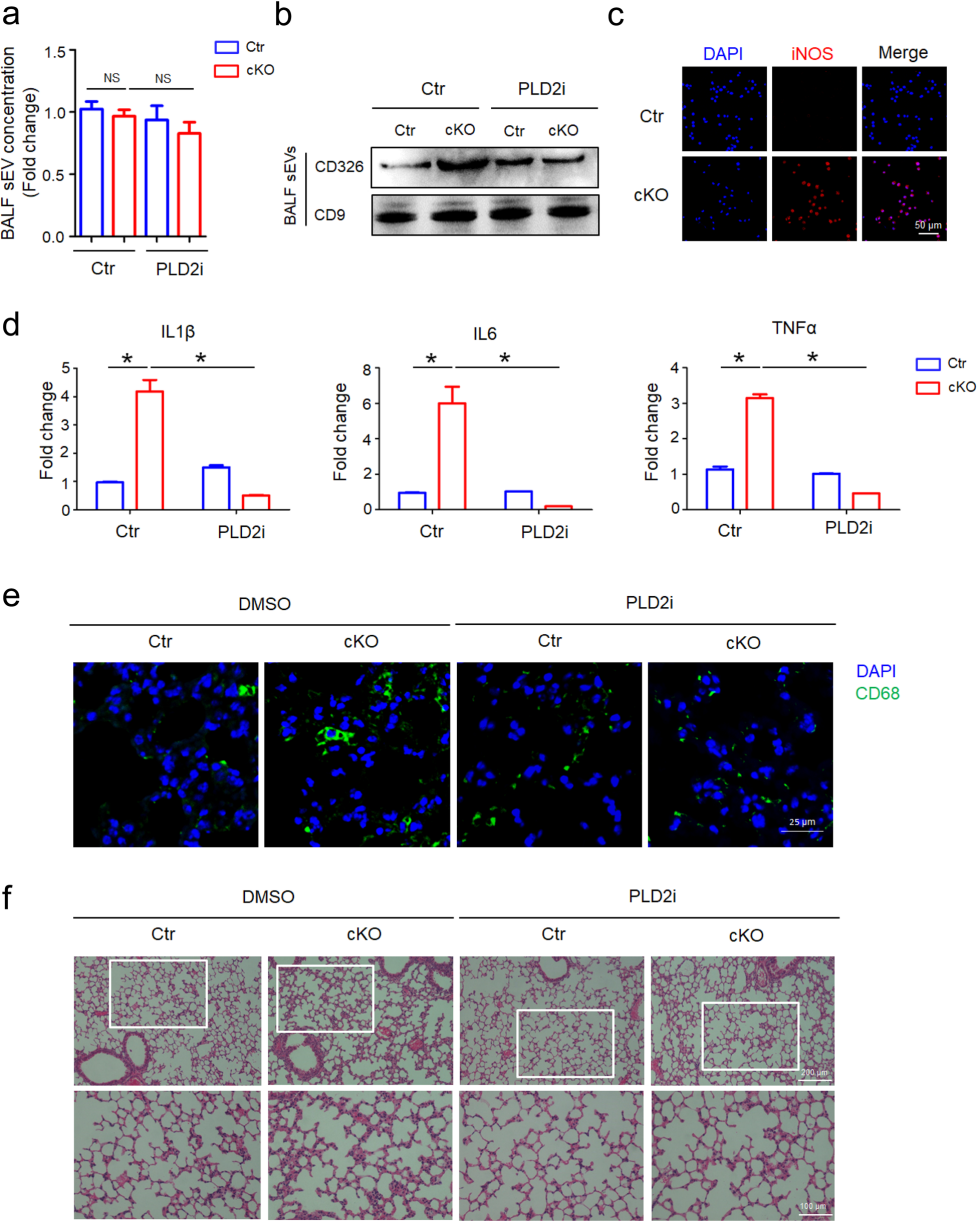
Increased epithelial sEVs facilitate macrophage activation and lung inflammation in vivo. [(a) DMSO or PLD2i (CAY10594, 2 mg/kg) was administrated to Ctr and cKO (ATII conditional Shp2 KO) mice. sEVs in the BALF were isolated for Nanosight analysis. Fold change is compared to control. (b) sEVs in the BALF were blotted for CD326 and CD9. (c) Levels of inducible nitric oxide synthase (iNOS) in alveolar macrophages in Ctr and cKO (ATII conditional Shp2 KO) mice. (d) The mRNA levels of inflammatory cytokine IL1β, IL6 and TNFα in alveolar macrophages were determined by qPCR. Fold change is compared to control. (e)CD68^+^ macrophages were examined in mouse lung specimens by immunofluorescence staining. (f) Haematoxylin and eosin (HE) stains of mouse lung sections. Data from three independent experiments are shown. *P < 0.05, **P < 0.01 and ***P < 0.001]

Therefore, we further investigated possible effects of Shp2 in vivo. First, we successfully isolated BALF (Bronchoalveolar lavage fluid) sEVs (Figure S11A and B). In ATII conditional Shp2‐KO mice, BALF sEVs were not changed significantly (Figure 7a). However, we observed upregulated sEV number indicated by epithelial marker CD326 (Figure 7b). It suggests that loss of Shp2 specifically in ATII cells resulted in enhanced production of epithelial sEVs in BALF. Then we isolated AMs (Alveolar macrophages) to study the effects of enhanced epithelial sEVs (Figure S11C). Compared with control, we found upregulated iNOS level in AMs, which were isolated from cKO group (Figure 7c). It suggests that enhanced production of epithelial sEVs induced M1 activation of AMs.

In ATII conditional Shp2‐KO mice, BALF AMs were collected and analyzed for inflammatory gene expressions. The results showed upregulated IL1β, IL6 and TNFα expression of AMs in cKO group (Figure 7d). We also observed enhanced pulmonary infiltration of macrophages (Figure 7e), higher levels of inflammatory cytokine in the lungs (Figure S11D) and inflammation‐associated alveolar damage (Figure 7f) compared with their littermates.

It is reported that Syntenin‐mediated sEV biogenesis and budding into multivesicular bodies are controlled by PLD2 (Ghossoub et al., 2014). In order to test whether this in vivo effect was induced by Shp2–Syntenin regulated epithelial sEVs, we administrated PLD2 inhibitor to Ctr and ATII conditional Shp2‐KO mice. As a result, PLD2 inhibitor reduced the epithelial sEVs (Figure 7b), inflammatory cytokine levels of AMs (Figure 7d), pulmonary infiltration of macrophages (Figure 7e), levels of inflammatory cytokine in the lungs (Figure S11D) and inflammation‐associated alveolar damage (Figure 7f) in ATII conditional Shp2‐KO mice compared with their littermates.

## 4 DISCUSSION

Extracellular vesicles (EVs) are defined as the generic term for cell‐released particles with a lipid bilayer and unable to replicate (Théry et al., 2018). The nanoscale EVs are released by most cell types and act as a mediator in intercellular communication.

EVs are divided into different types according to the descriptions of conditions: apoptosomes, which are released from apoptotic cells; microvesicles, which are shed by evagination of the plasma membrane of healthy cells; and exosomes, which are ILVs released into the extracellular environment by the fusion of MVBs with the plasma membrane (Raposo & Stoorvogel, 2013; Song et al., 2019). In addition, EVs are also classified as small EVs (< 200 nm) and medium/large EVs (> 200 nm), depending on physical sizes (Théry et al., 2018). In the present study, we isolated small EVs by sequential centrifugation and filtration as previously described (Moroishi et al., 2016): centrifugations at low speeds, followed by filtration through 0.22 μm PVDF filter and ultracentrifugation at 110,000 × *g* for 90 min. It is a widely used protocol for exosome isolation and the pellet was enriched with exosomes (Lobb et al., 2015). However, it probably also contained other vesicle populations of similar sizes (Song et al., 2019). Moreover, the term ‘exosomes’ was first described as transforming DNA fragments and still used today (Mishra & Tatum, 1973). ‘Extracellular vesicle’ rather than ‘exosome’ was first used to describe EVs (Aaronson et al., 1971). To avoid confusion and inconsistent usages as previously presented (Witwer & Théry, 2019), we used the term ‘small EVs’ rather than ‘exosomes’ in this study.

**FIGURE 8:**
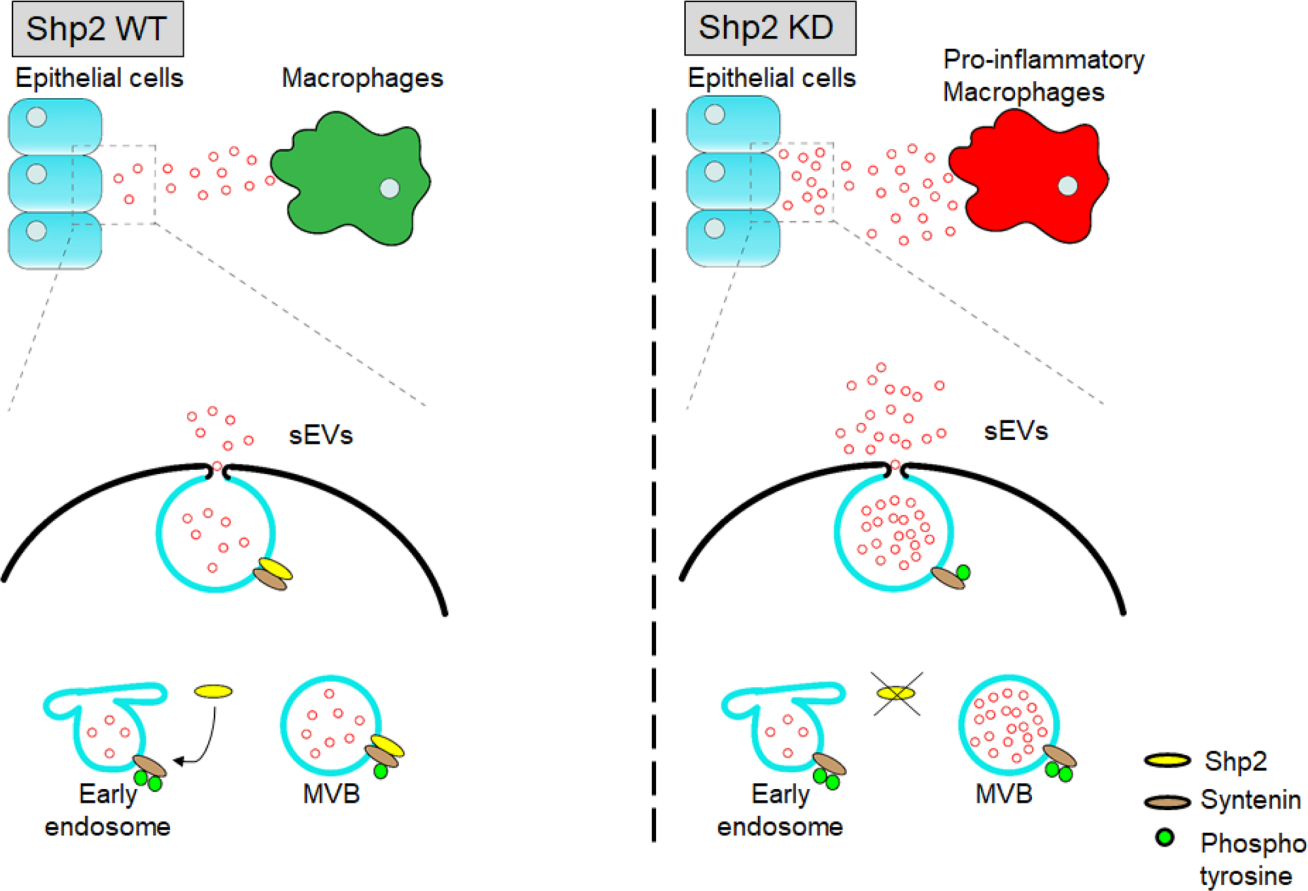
A schematic representation of a model for the role of Shp2 in the regulation of sEV biogenesis. [Shp2 decreases the tyrosine phosphorylation of Syntenin. Shp2 depletion increased Syntenin‐mediated sEV formation, thus promoting sEVs transferring from epithelial cells to macrophages. As a result, increased epithelial sEVs induced macrophages activation]

The sEV‐mediated intercellular communication is a complex biological process and requires precise regulation of multiple signalling pathways. Despite increasing progress identifying several critical regulators, little is known about the negative controllers in sEV biogenesis and secretion. It is reported that many kinases promote the generation of sEVs by phosphorylating downstream signal proteins (Dautova et al., 2018; Hirsova et al., 2016; Lee et al., 2018; Wei et al., 2017; Zhang et al., 2018). Comparatively, phosphatases are the key enzymes characterized by removing phosphatase groups and reversing the effect of kinase‐directed phosphorylation. Therefore, we investigated the role of phosphatases in sEV formation. In this present study, we identified the function of tyrosine phosphatase Shp2 as a negative regulator of sEV biogenesis. Our results showed that Shp2 depletion dramatically increased sEV production both in vitro and in vivo, which was accompanied by upregulation of the tyrosine phosphorylation of Syntenin, an adaptor protein reported to regulate ILV biogenesis in MVBs. The elevated level of sEV number in the absence of Shp2 was due to upregulated tyrosine phosphorylation of Syntenin. Furthermore, as in vitro dephosphorylation assays indicated, purified recombinant Shp2 directly dephosphorylated Syntenin Y46. Our data indicated that Shp2 negatively controlled sEV numbers rather properties in epithelial cells. In vitro sEV transfer model showed increased sEV transfer from epithelial cells to macrophages by epithelial Shp2 stable KD. The increase of epithelial sEVs caused by shRNA‐mediated down‐regulation of Shp2 promoted macrophage activation, resulting in enhanced inflammation. In summary, as a regulator of Syntenin Y46 phosphorylation, Shp2 negatively controls sEV biogenesis in vitro and in vivo. Moreover, Shp2 regulates the inflammatory effect of increased epithelial sEVs on macrophages (Figure 8).

Syntenin is highly enriched in sEVs and plays an important role in the formation of sEVs (Ghossoub et al., 2014). Syntenin binds to the auxiliary ESCRT component ALIX through its N‐terminal domain and participates in the budding of sEVs (Baietti et al., 2012). Syntenin's PDZ domain directly binds to a variety of membrane receptors such as Syndecan and tetraspanin, thereby participating in the generation of sEVs (Latysheva et al., 2006). Moreover, Syntenin directly interacts with the auxiliary component ALIX of ESCRT through the N‐terminal LYPXnL motifs, and binds to the conserved intracellular segment of Syndecan through the PDZ domain (Baietti et al., 2012), thereby forming Syndecan‐Syntenin‐ALIX Complex. Because ALIX interacts with many ESCRT proteins, the Syntenin‐ALIX complex packages Syndecan‐mediated cargo into the ILVs of MVBs through the ESCRT mechanism (Hurley & Odorizzi, 2012). Syndecan‐mediated cargo is mainly bound to Syndecan's heparin sulfate. The cargo creates Syndecan assemblies and recruits Syntenin‐ALIX, subsequently supporting membrane budding. In this process, heparanase cleaves heparin sulfate to promote the secretion of sEVs (Roucourt et al., 2015). In addition, Syntenin also binds to the small GTPase ARF6, and promotes the generation of sEVs through its downstream signalling molecule PLD2 (Ghossoub et al., 2014). Recent studies find that kinase Src phosphorylates Syntenin and activates the signalling pathway of Syntenin‐mediated sEV production, in which the phosphorylation of tyrosine at the Y46 site of Syntenin functions as a molecular switch (Imjeti et al., 2017). In the present study, we found that tyrosine phosphatase Shp2 negatively controlled sEV formation by dephosphorylating Syntenin Y46. sEV‐controlled intercellular contacts influence homeostasis in multicellular organisms (Hessvik & Llorente, 2018; Stahl & Raposo, 2019). The mechanism of sEV biogenesis has critical role in regulating homeostasis of sEV formation and release. As a molecular brake of sEV biogenesis, Shp2 was found to control homeostasis of sEV production, thus regulating the balance of cell‐cell crosstalk. Moreover, it was shown that Shp2 regulated sEV number other than components in epithelial cells. However, recent studies indicate that PDGFRα is enriched in EVs derived from PDGF‐BB‐treated HSCs (Hepatic stellate cells) in an Shp2‐dependent manner (Kostallari et al., 2018). In vitro, PDGF and its downstream molecule Shp2 increase HSC‐derived EV release. Shp2 inhibition abolishes PDGF‐mediated EV release (Gao et al., 2020). In these investigations, Shp2 acts as a downstream molecule activated by PDGF rather than an inherent controller of EV formation. Besides, these studies investigate the role of Shp2 only in HSCs. So, it is possible that specific stimulation and cell specificity enable the role of Shp2 in promoting HSC‐EV release and mediating pro‐fibrotic cargo packaging. In addition, it is not further studied whether Shp2 regulates HSC‐EVs with the help of previously reported regulators involved in EV biogenesis. In order to clarify the overall function of Shp2 in sEV biogenesis, further investigations are necessary to study the underlying mechanisms in various cells or under different stimulation.

Lungs, as the respiratory organs of the human body, are susceptible to external stimulation and suffer from various diseases. The alveolar sacs are at the distal end of respiratory system. As the smallest respiratory unit, alveoli are damaged in many pulmonary diseases. The alveolar microenvironment is mainly composed of alveolar epithelial cells, alveolar macrophages, and pulmonary microvascular endothelial cells. During inflammation, neutrophils also infiltrate into the alveoli, forming a complex microenvironment (Thompson et al., 2017). Researches show that sEVs in the alveolar microenvironment regulate lung inflammation and injury processes, thereby affecting various disease pathogenesis including acute lung injury and chronic obstructive pulmonary disease (Fujita et al., 2015). In the process of injury and inflammation, alveolar macrophages are the important defenders, regulating the disease process through their activation. Alveolar epithelial cells form the basic structure of the alveolar cavity, which plays a critical role in maintaining the alveolar morphology and physiological function, and resisting various external stimuli (Lee et al., 2018). Increasing literatures show that sEV‐mediated communication between alveolar epithelium and alveolar macrophages is involved in regulating the development of lung diseases (Haggadone & Peters‐Golden, 2018; Lee et al., 2018). In acute lung injury, sEV transmission between epithelial cells and macrophages regulates the inflammation and injury process (Lee et al., 2018). In acute lung injury induced by hyperoxia, sEVs derived from alveolar epithelial cells activate alveolar macrophages through the ROCK1 pathway, thereby aggravating the disease process (Moon et al., 2015). In asthma, sEVs derived from lung epithelium induce the proliferation and chemotaxis of undifferentiated macrophages, thereby exacerbating the pathological indicators of asthma (Kulshreshtha et al., 2013). Analyzing the pattern of sEV transmission between alveolar epithelium and alveolar macrophages provides new vision for the study and treatment of lung diseases. In the study, we established an indirect co‐culture system for in vitro donor–acceptor sEV transfer, thereby facilitating the investigations of sEV transfer in alveolar microenvironment. Our findings provide a basis for understanding the mechanism of sEV formation and its function in epithelial‐macrophage crosstalk. The interaction of Shp2 with Syntenin in controlling sEV biogenesis is supposed to be involved in the initiation and progression of pulmonary diseases. Our study elucidates the new function of Shp2 in the alveolar microenvironment by controlling sEV biogenesis.

Taken together, the above findings identify Shp2‐Syntenin as an important regulator of sEV biogenesis. By extension, disruption of this axis also influences epithelial‐macrophage crosstalk in alveolar microenvironment. sEVs play a key role in various cell functions, so the underlying molecular mechanism of sEV biogenesis is worth further exploration.

## CONFLICTS OF INTEREST

The authors declare no conflict of interest.

## ETHICS STATEMENT

All animal protocols and experiments for this study were approved by the Institutional Animal Care and Use Committee of Zhejiang University School of Medicine (No.ZJU20200167).

## FINANCIAL SUPPORT

This work was supported by National Natural Science Foundation of China (81530001 and 81873418 to Y. Ke; 31870901to X. Zhang; 81800459 to J. Xu), Key Research and Development Project of Ministry of Science and Technology of China (2016YFA0501800 to Y. Ke).

## Supporting information

Supporting Information
**Figure S1**. (A)Phosphatase inhibitor treatment on sEV secretion of MLE‐12 cells. SHP099 10 μM (Shp2 inhibitor), SOV 10 μM (Sodium orthovanadate, tyrosine phosphatase inhibitor), VO‐Ohpic 2.5 μM (PTEN inhibitor), PTP1B‐IN‐2 5 μM (PTP1B inhibitor). (B)Phosphatase inhibitor treatment on sEV secretion of Jurkat cells and BMDM. NQ301 0.8 μM (CD45 inhibitor), Etoposide 2 μM (TCPTP inhibitor), TPI‐1 8 μM (Shp1 inhibitor).
**Figure S2**. After treatment of inhibitors for 24 h, the cell viability was measured by CCK‐8 assay. MLE‐12 cells: SHP099 (Shp2 inhibitor), SOV (Sodium orthovanadate, tyrosine phosphatase inhibitor), VO‐Ohpic (PTEN inhibitor), CAY10594 (PLD2 inhibitor). Jurkat cells: NQ301 (CD45 inhibitor), Etoposide (TCPTP inhibitor). BMDM: TPI‐1 (Shp1 inhibitor).
**Figure S3**. (A)Western blot analysis of Shp2 level in siShp2 BMDM and shShp2 MCF‐7, compared with control. (B)For BMDM, sEV numbers of Shp2i (Shp2 inhibitor, SHP099 10 μM) and Shp2 KO group were counted by NanoSight quantification. sEV concentration represents the quantity of sEVs released by same number of cells. Fold change is compared to control. For MCF‐7, Shp2i (Shp2 inhibitor, SHP099 20 μM) was conducted as above mentioned.
**Figure S4**. (A)Genotyping analysis was performed with mouse tail genomic DNA by PCR. (B)Confocal micrographs show Shp2 level in ATII cells of Ctr and cKO (ATII conditional Shp2 KO) mice. Data from three independent experiments are shown.
**Figure S5**. MS analysis of sEVs in shScr and shShp2 stable epithelial cell lines (MLE‐12 cells). sEVs for MS were purified from cell culture supernatants from shScr and shShp2 stable epithelial cell lines (MLE‐12 cells).
**Figure S6**. Analysis of sEVs by nanoscale flow cytometry using indicated antibodies. sEVs were purified from cell culture supernatants from shScr and shShp2 stable epithelial cell lines (MLE‐12 cells). Data from three independent experiments are shown.
**Figure S7**. MS analysis of total proteins in shScr and shShp2 stable epithelial cell lines (MLE‐12 cells). Proteins are arranged according to fold change values. Upregulated and downregulated proteins are indicated by red and green hues, respectively.
**Figure S8**. MS analysis of proteins involved in sEV biogenesis in the shScr and shShp2 stable epithelial cell lines (MLE‐12 cells). Proteins are arranged according to fold change values.
**Figure S9**. (A)Western blot analysis of shScr and shShp2 stable epithelial cell lines (MLE‐12 cells). 293T cells were cotransfected with Shp2‐Myc and CD9‐Flag (B), ALIX‐Flag (C), Flotillin 1 ‐Flag (D), YKT6‐Flag (E) respectively. The cells were lysed and immunoprecipitated (IP) using the indicated antibody or IgG. The interaction between Shp2‐Myc and the mentioned protein was detected by western blot with the indicated antibody. (F) Western blot analysis of Syntenin in siSyntenin MLE‐12 cells.
**Figure S10**. Schematic illustration of indirect co‐culture system for in vitro donoracceptor sEV transfer.
**Figure S11**. (A)Negatively stained TEM image of purified sEVs from BALF. (B)Western blot analysis of sEVs purified from BALF. Lung tissue and sEVs were blotted for ALIX, TSG101, CD9, GM130, Calnexin and GRP94. (C)Confocal micrographs show alveolar macrophages (indicated by CD68) isolated from mouse lung. (D)DMSO or PLD2i (CAY10594, 2 mg/Kg) was administrated to Ctr and cKO (ATII conditional Shp2 KO) mice. The mRNA levels of inflammatory cytokine IL1β, IL6 and TNFα in lung tissue were determined by qPCR. Fold change is compared to control.

## Data Availability

The data that support the findings of this study are available from the corresponding author upon reasonable request.
