# Supplementary material for: Phosphatase Shp2 regulates biogenesis of small extracellular vesicles by dephosphorylating Syntenin
Source: J Extracell Vesicles. 2021 Mar 10;10(5):e12078. doi: 10.1002/jev2.12078 (PMC7944561; doi:10.1002/jev2.12078)
Supplement: Supplementary file 1 — Supporting Information Figure S1. (A)Phosphatase inhibitor treatment on sEV secretion of MLE‐12 cells. SHP099 10 μM (Shp2 inhibitor), SOV 10 μM (Sodium orthovanadate, tyrosine phosphatase inhibitor), VO‐Ohpic 2.5 μM (PTEN inhibitor), PTP1B‐IN‐2 5 μM (PTP1B inhibitor). (B)Phosphatase inhibitor treatment on sEV secretion of Jurkat cells and BMDM. NQ301 0.8 μM (CD45 inhibitor), Etoposide 2 μM (TCPTP inhibitor), TPI‐1 8 μM (Shp1 inhibitor). Figure S2. After treatment of inhibitors for 24 h, the cell viability was measured by CCK‐8 assay. MLE‐12 cells: SHP099 (Shp2 inhibitor), SOV (Sodium orthovanadate, tyrosine phosphatase inhibitor), VO‐Ohpic (PTEN inhibitor), CAY10594 (PLD2 inhibitor). Jurkat cells: NQ301 (CD45 inhibitor), Etoposide (TCPTP inhibitor). BMDM: TPI‐1 (Shp1 inhibitor). Figure S3. (A)Western blot analysis of Shp2 level in siShp2 BMDM and shShp2 MCF‐7, compared with control. (B)For BMDM, sEV numbers of Shp2i (Shp2 inhibitor, SHP099 10 μM) and Shp2 KO group were counted by NanoSight quantification. sEV concentration represents the quantity of sEVs released by same number of cells. Fold change is compared to control. For MCF‐7, Shp2i (Shp2 inhibitor, SHP099 20 μM) was conducted as above mentioned. Figure S4. (A)Genotyping analysis was performed with mouse tail genomic DNA by PCR. (B)Confocal micrographs show Shp2 level in ATII cells of Ctr and cKO (ATII conditional Shp2 KO) mice. Data from three independent experiments are shown. Figure S5. MS analysis of sEVs in shScr and shShp2 stable epithelial cell lines (MLE‐12 cells). sEVs for MS were purified from cell culture supernatants from shScr and shShp2 stable epithelial cell lines (MLE‐12 cells). Figure S6. Analysis of sEVs by nanoscale flow cytometry using indicated antibodies. sEVs were purified from cell culture supernatants from shScr and shShp2 stable epithelial cell lines (MLE‐12 cells). Data from three independent experiments are shown. Figure S7. MS analysis of total proteins in shScr and shShp2 stable [file JEV2-10-e12078-s001.pdf]

Figure S1

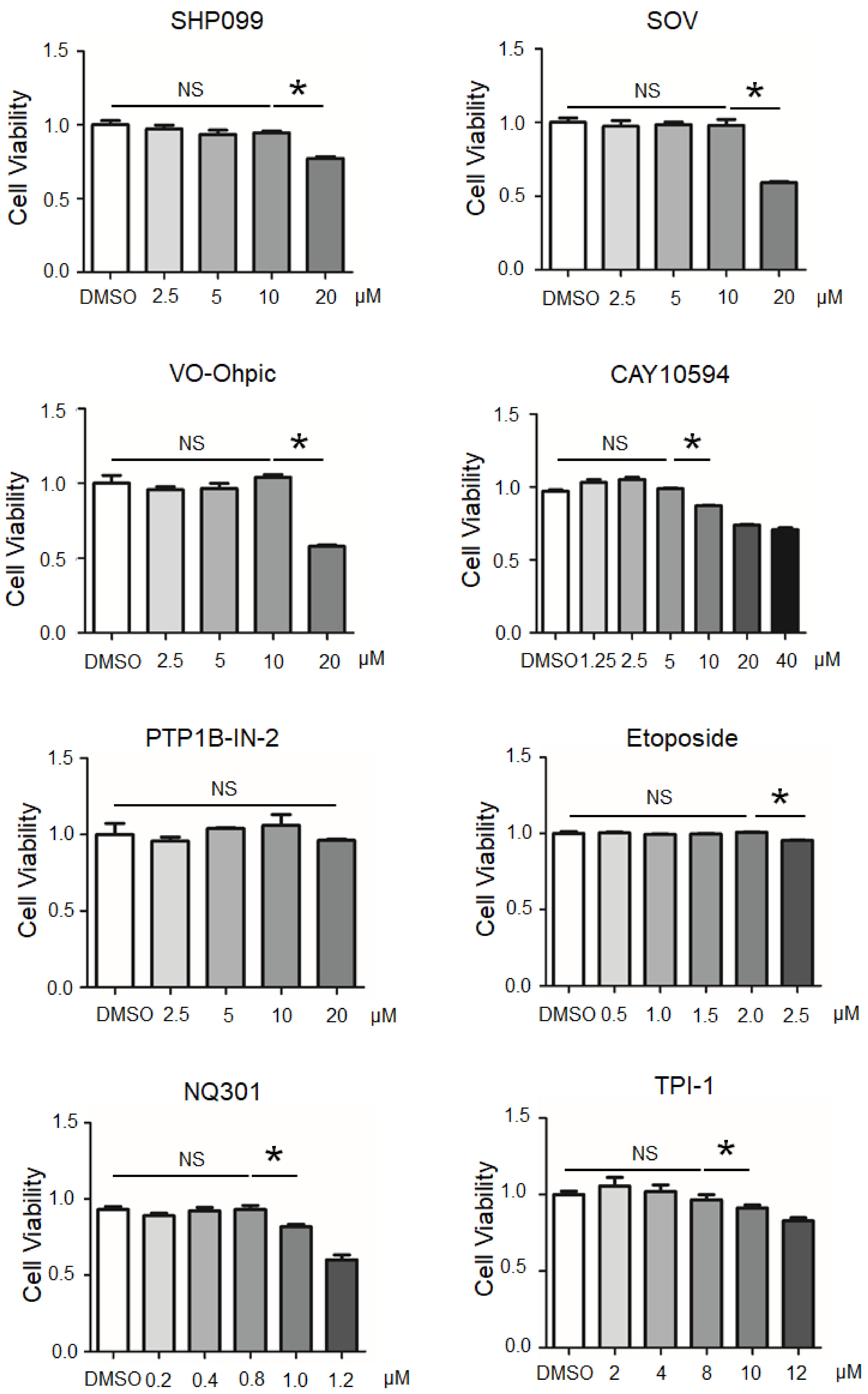
SHP099
Cell Viability
1.5
1.0
0.5
0.0
NS
*
DMSO 2.5 5 10 20 μM
SOV
Cell Viability
1.5
1.0
0.5
0.0
NS
*
DMSO 2.5 5 10 20 μM
VO-Ohpic
Cell Viability
1.5
1.0
0.5
0.0
NS
*
DMSO 2.5 5 10 20 μM
CAY10594
Cell Viability
1.5
1.0
0.5
0.0
NS
*
DMSO 1.25 2.5 5 10 20 40 μM
PTP1B-IN-2
Cell Viability
1.5
1.0
0.5
0.0
NS
DMSO 2.5 5 10 20 μM
Etoposide
Cell Viability
1.5
1.0
0.5
0.0
NS
*
DMSO 0.5 1.0 1.5 2.0 2.5 μM
NQ301
Cell Viability
1.5
1.0
0.5
0.0
NS
*
DMSO 0.2 0.4 0.8 1.0 1.2 μM
TPI-1
Cell Viability
1.5
1.0
0.5
0.0
NS
*
DMSO 2 4 8 10 12 μM

Figure S2

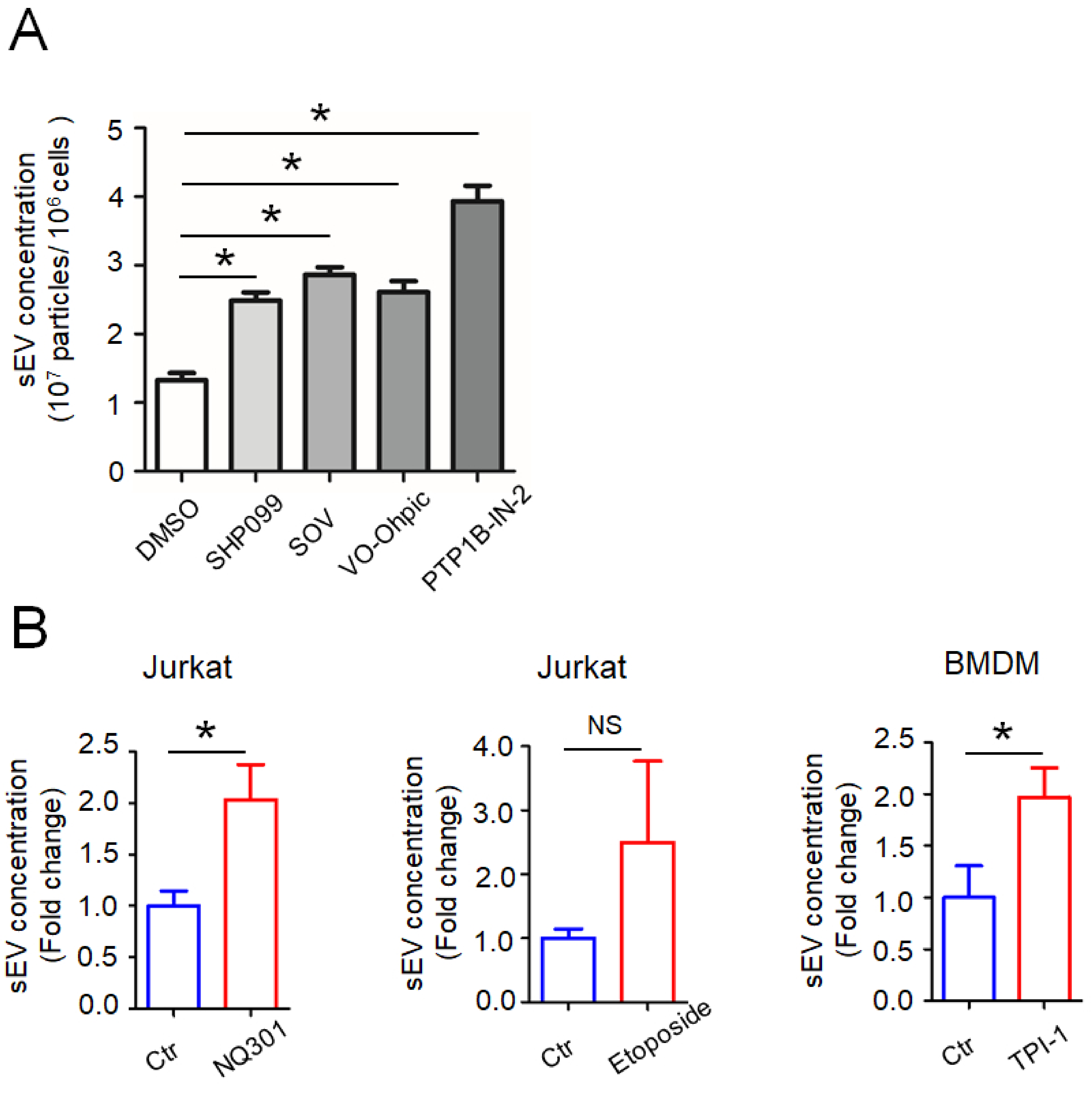
A
sEV concentration
(10^7 particles/ 10^6 cells)
5
4
3
2
1
0
*
*
*
*
DMSO
SHP099
SOV
VO-Ohpic
PTP1B-IN-2
B
Jurkat
sEV concentration
(Fold change)
2.5
2.0
1.5
1.0
0.5
0.0
*
Ctr
NQ301
Jurkat
sEV concentration
(Fold change)
4.0
3.0
2.0
1.0
0.0
NS
Ctr
Etoposide
BMDM
sEV concentration
(Fold change)
2.5
2.0
1.5
1.0
0.5
0.0
*
Ctr
TPI-1

Figure S3

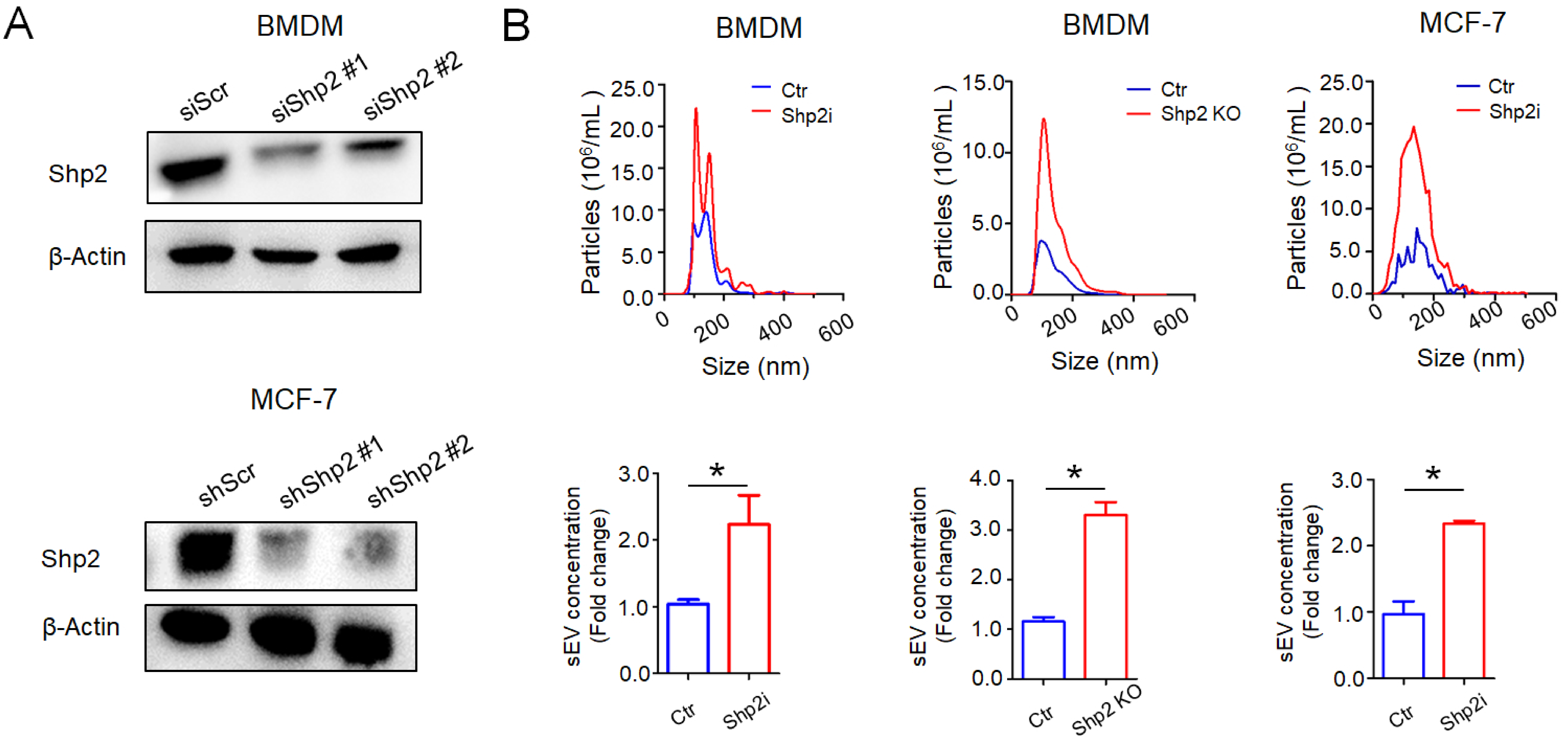
A
BMDM
siScr
siShp2 #1
siShp2 #2
Shp2
β-Actin
MCF-7
shScr
shShp2 #1
shShp2 #2
Shp2
β-Actin
B
BMDM
Particles (10^6/mL)
Ctr
Shp2i
Size (nm)
BMDM
Ctr
Shp2 KO
Size (nm)
MCF-7
Ctr
Shp2i
Size (nm)
sEV concentration (Fold change)
*
Ctr
Shp2i
Ctr
Shp2 KO
Ctr
Shp2i

Figure S4

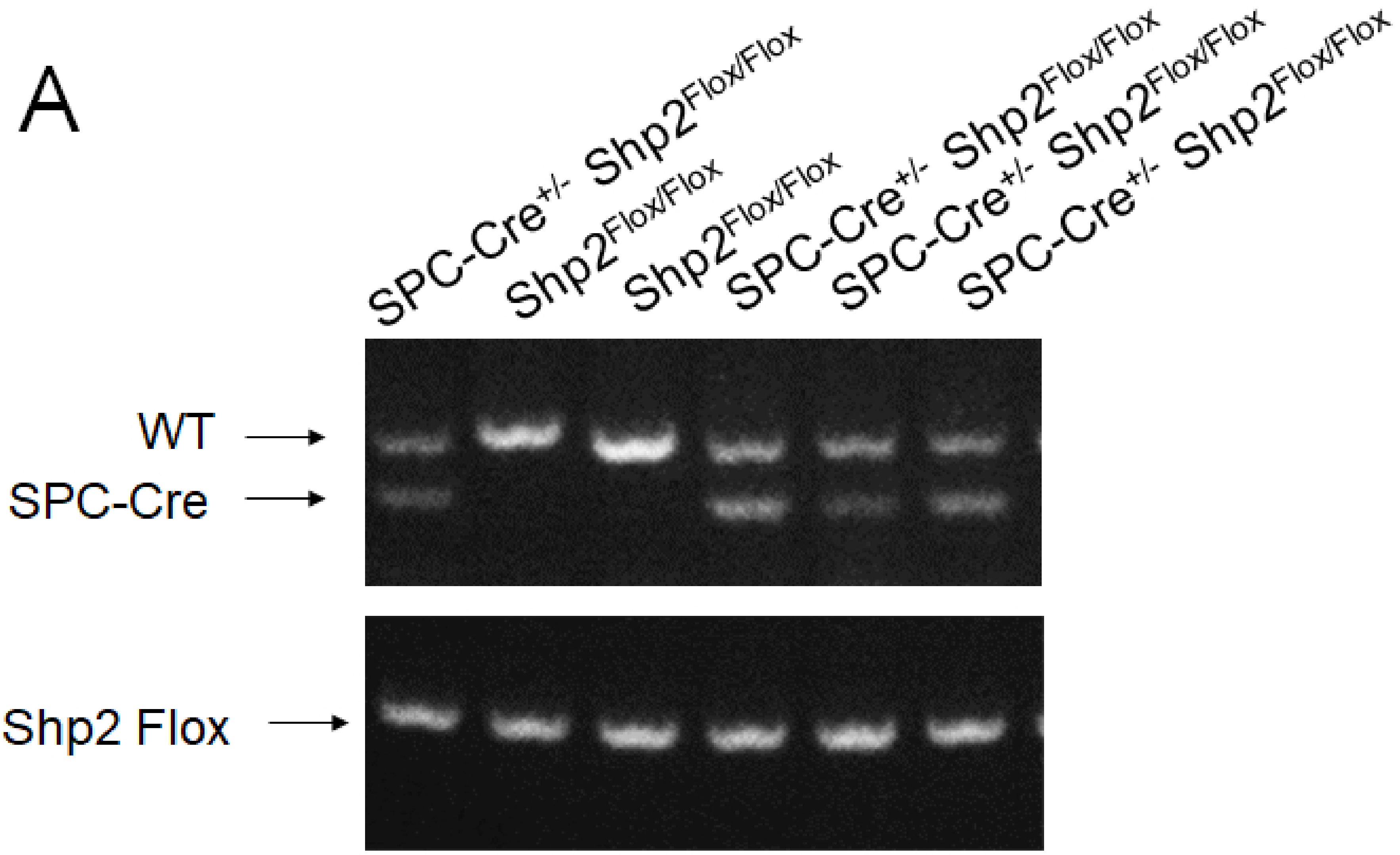
A
SPC-Cre+/- Shp2Flox/Flox
Shp2Flox/Flox
Shp2Flox/Flox
SPC-Cre+/- Shp2Flox/Flox
SPC-Cre+/- Shp2Flox/Flox
SPC-Cre+/- Shp2Flox/Flox
WT
SPC-Cre
Shp2 Flox

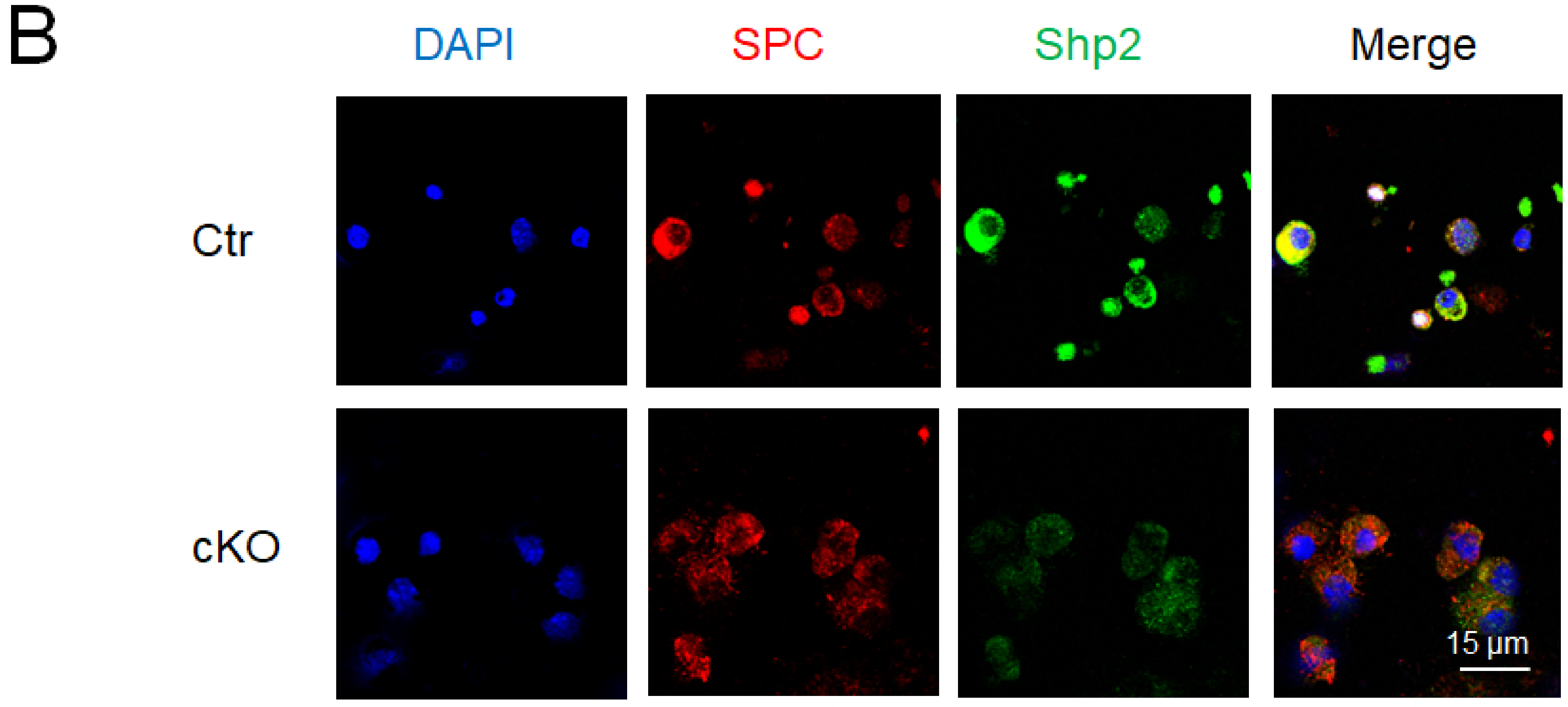
B
DAPI
SPC
Shp2
Merge
Ctr
cKO
15 µm

Figure S5

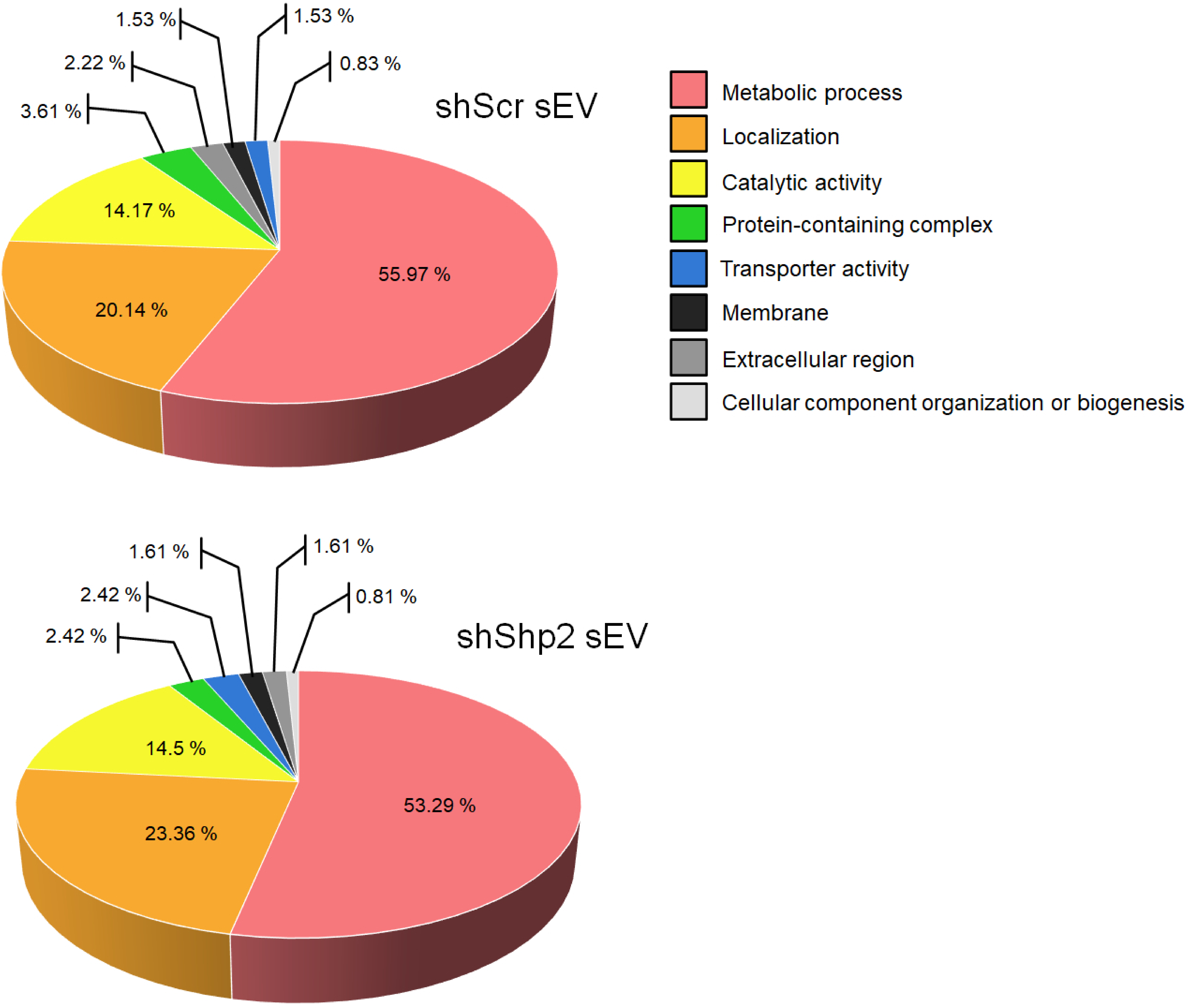
shScr sEV
55.97 %
20.14 %
14.17 %
3.61 %
2.22 %
1.53 %
1.53 %
0.83 %
Metabolic process
Localization
Catalytic activity
Protein-containing complex
Transporter activity
Membrane
Extracellular region
Cellular component organization or biogenesis
shShp2 sEV
53.29 %
23.36 %
14.5 %
2.42 %
2.42 %
1.61 %
1.61 %
0.81 %

Figure S6

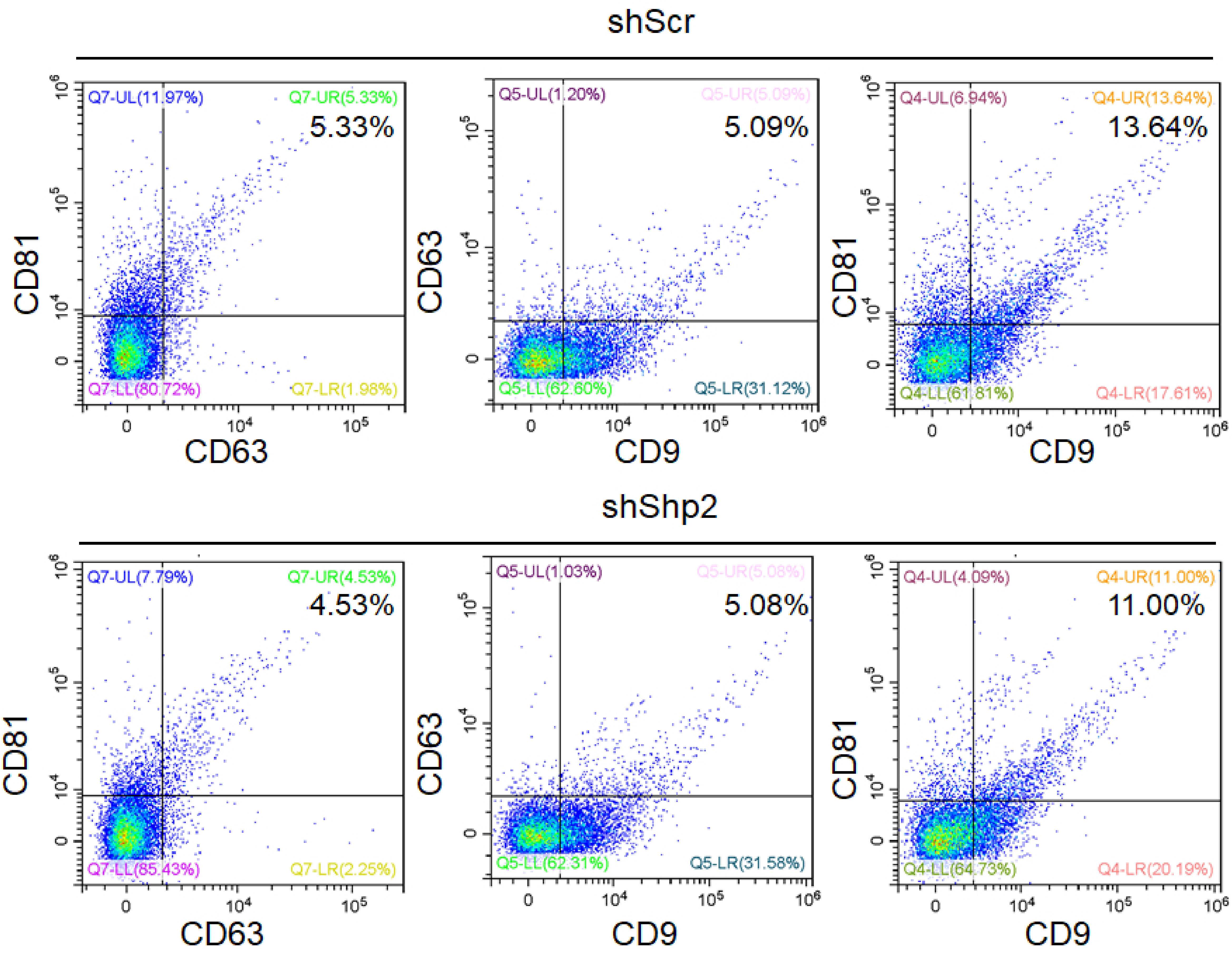

shScr
Q7-UL(11.97%)
Q7-UR(5.33%)
5.33%
Q7-LL(80.72%)
Q7-LR(1.98%)
CD81
CD63
Q5-UL(1.20%)
Q5-UR(5.09%)
5.09%
Q5-LL(62.60%)
Q5-LR(31.12%)
CD63
CD9
Q4-UL(6.94%)
Q4-UR(13.64%)
13.64%
Q4-LL(61.81%)
Q4-LR(17.61%)
CD81
CD9
shShp2
Q7-UL(7.79%)
Q7-UR(4.53%)
4.53%
Q7-LL(85.43%)
Q7-LR(2.25%)
CD81
CD63
Q5-UL(1.03%)
Q5-UR(5.08%)
5.08%
Q5-LL(62.31%)
Q5-LR(31.58%)
CD63
CD9
Q4-UL(4.09%)
Q4-UR(11.00%)
11.00%
Q4-LL(64.73%)
Q4-LR(20.19%)
CD81
CD9

Figure S7

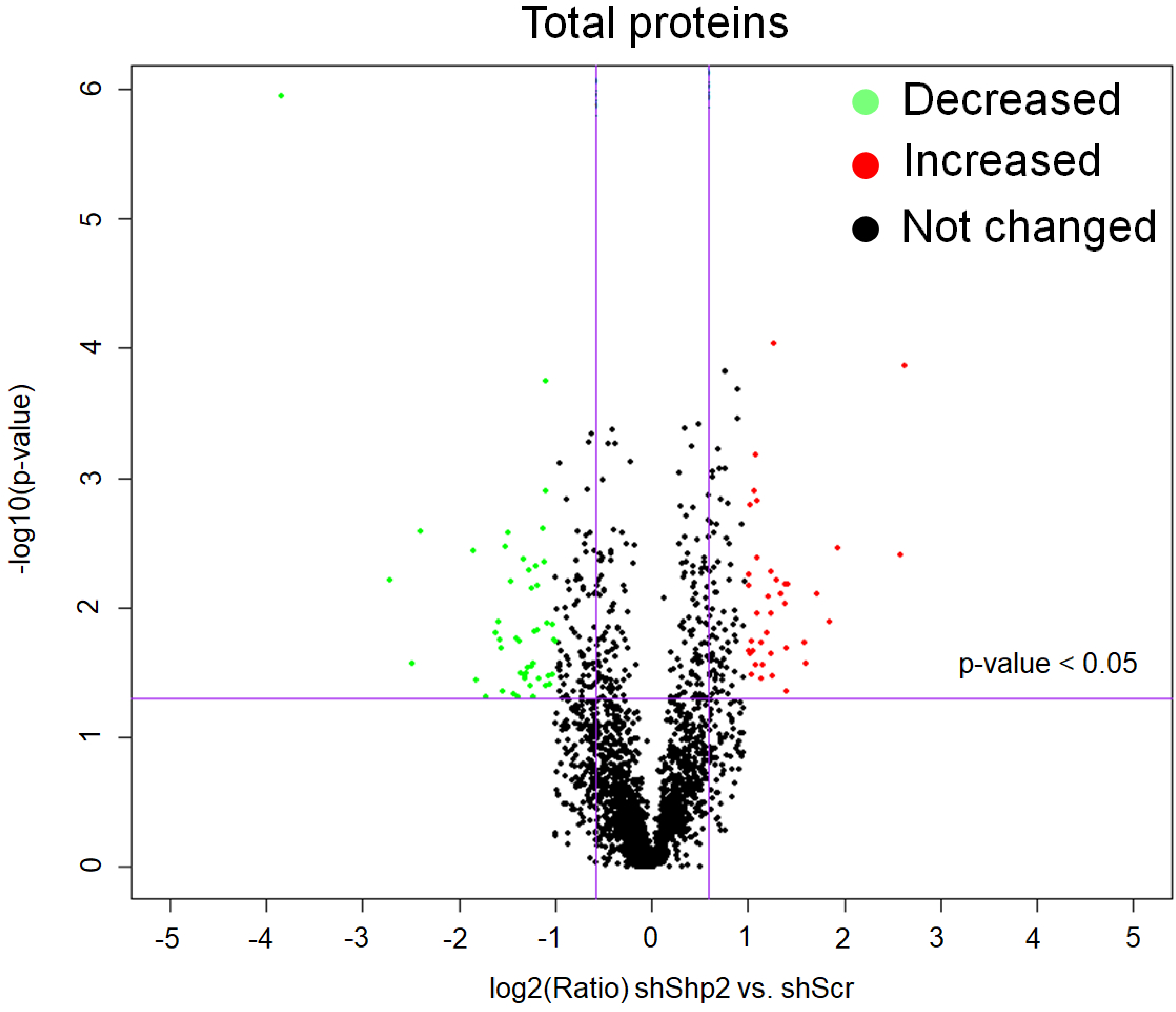

Total proteins
Decreased
Increased
Not changed
p-value < 0.05
-log10(p-value)
0
1
2
3
4
5
6
-5
-4
-3
-2
-1
0
1
2
3
4
5
log2(Ratio) shShp2 vs. shScr

Figure S8

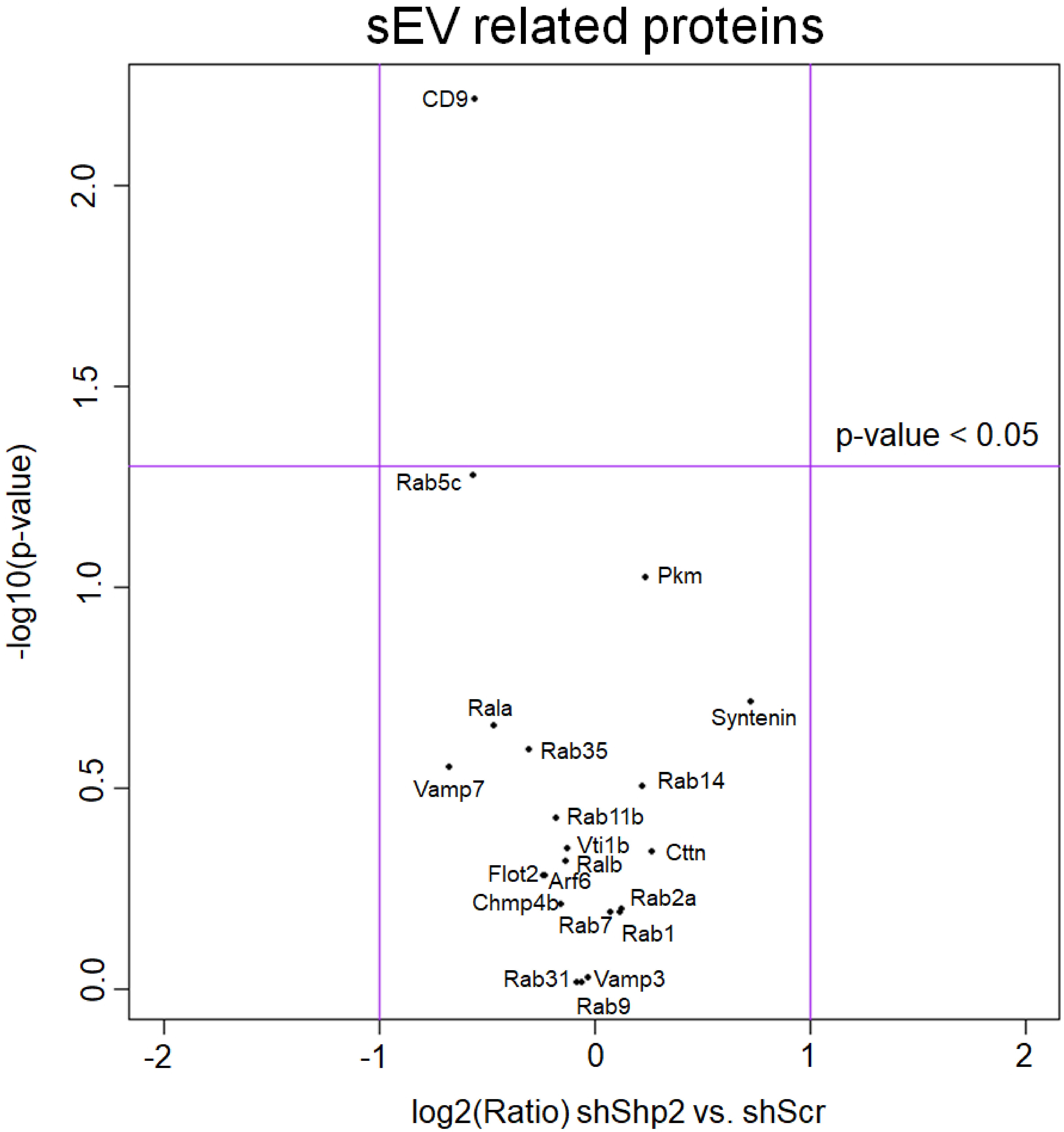

sEV related proteins
CD9
Rab5c
Pkm
Rala
Syntenin
Rab35
Vamp7
Rab14
Rab11b
Vti1b
Cttn
Ralb
Flot2
Arf6
Chmp4b
Rab2a
Rab7
Rab1
Rab31
Vamp3
Rab9
p-value < 0.05
-log10(p-value)
log2(Ratio) shShp2 vs. shScr
0.0
0.5
1.0
1.5
2.0
-2
-1
0
1
2

Figure S9

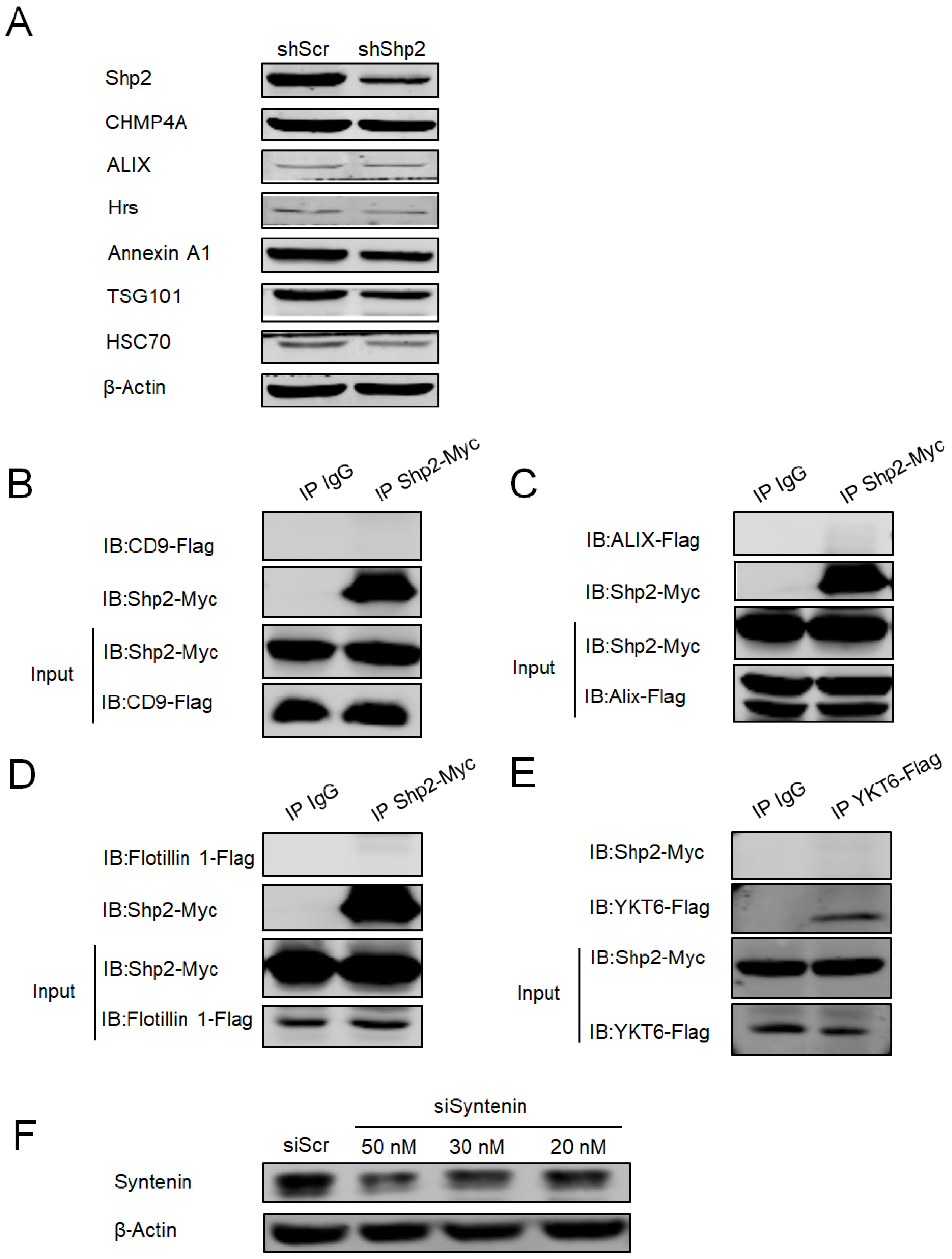
A
shScr
shShp2
Shp2
CHMP4A
ALIX
Hrs
Annexin A1
TSG101
HSC70
β-Actin
B
IP IgG
IP Shp2-Myc
IB:CD9-Flag
IB:Shp2-Myc
Input
IB:Shp2-Myc
IB:CD9-Flag
C
IP IgG
IP Shp2-Myc
IB:ALIX-Flag
IB:Shp2-Myc
Input
IB:Shp2-Myc
IB:Alix-Flag
D
IP IgG
IP Shp2-Myc
IB:Flotillin 1-Flag
IB:Shp2-Myc
Input
IB:Shp2-Myc
IB:Flotillin 1-Flag
E
IP IgG
IP YKT6-Flag
IB:Shp2-Myc
IB:YKT6-Flag
Input
IB:Shp2-Myc
IB:YKT6-Flag
F
siSyntenin
siScr
50 nM
30 nM
20 nM
Syntenin
β-Actin

Figure S10

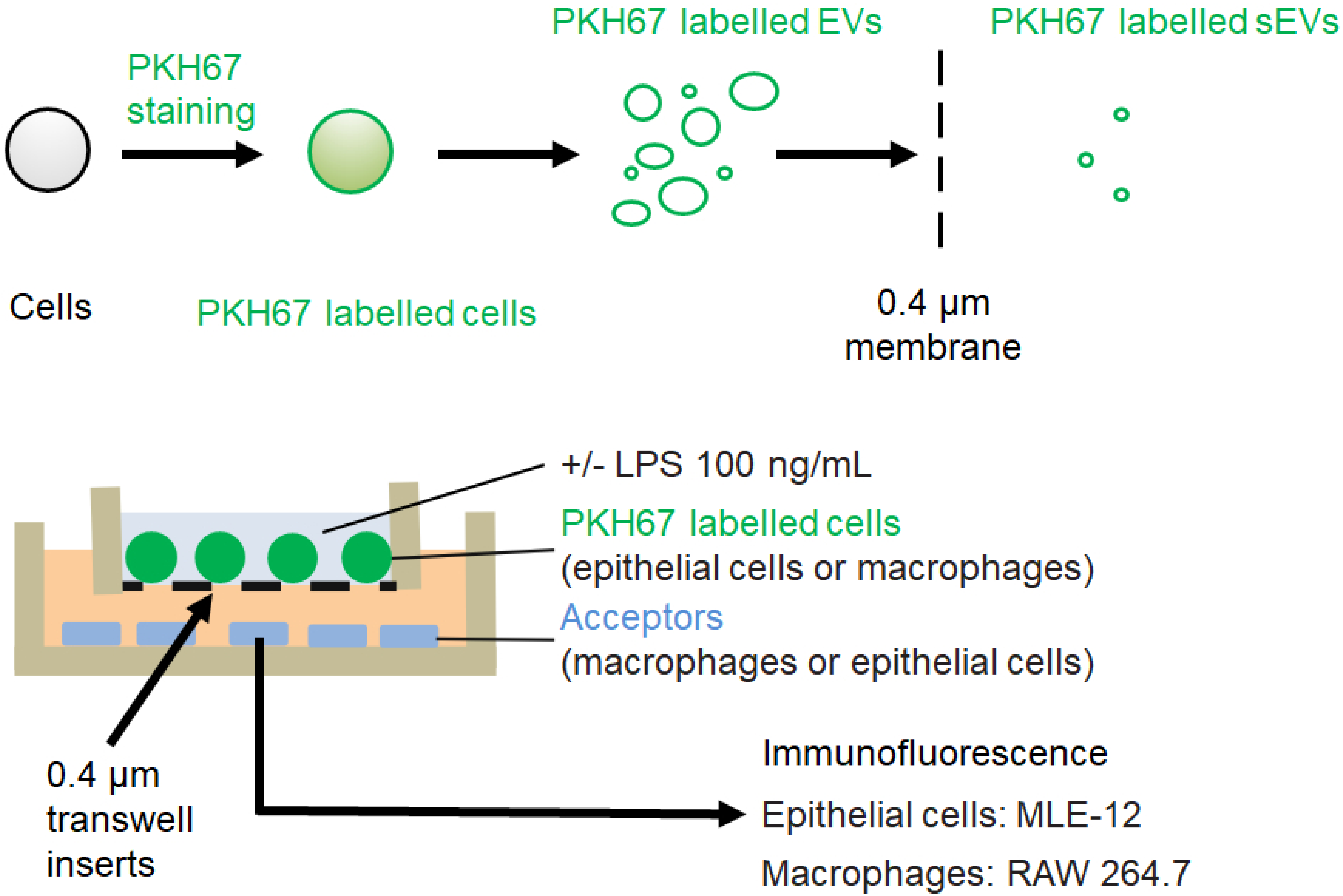

PKH67 labelled EVs
PKH67 labelled sEVs
PKH67 staining
Cells
PKH67 labelled cells
0.4 µm membrane
+/- LPS 100 ng/mL
PKH67 labelled cells
(epithelial cells or macrophages)
Acceptors
(macrophages or epithelial cells)
Immunofluorescence
0.4 µm transwell inserts
Epithelial cells: MLE-12
Macrophages: RAW 264.7

Figure S11

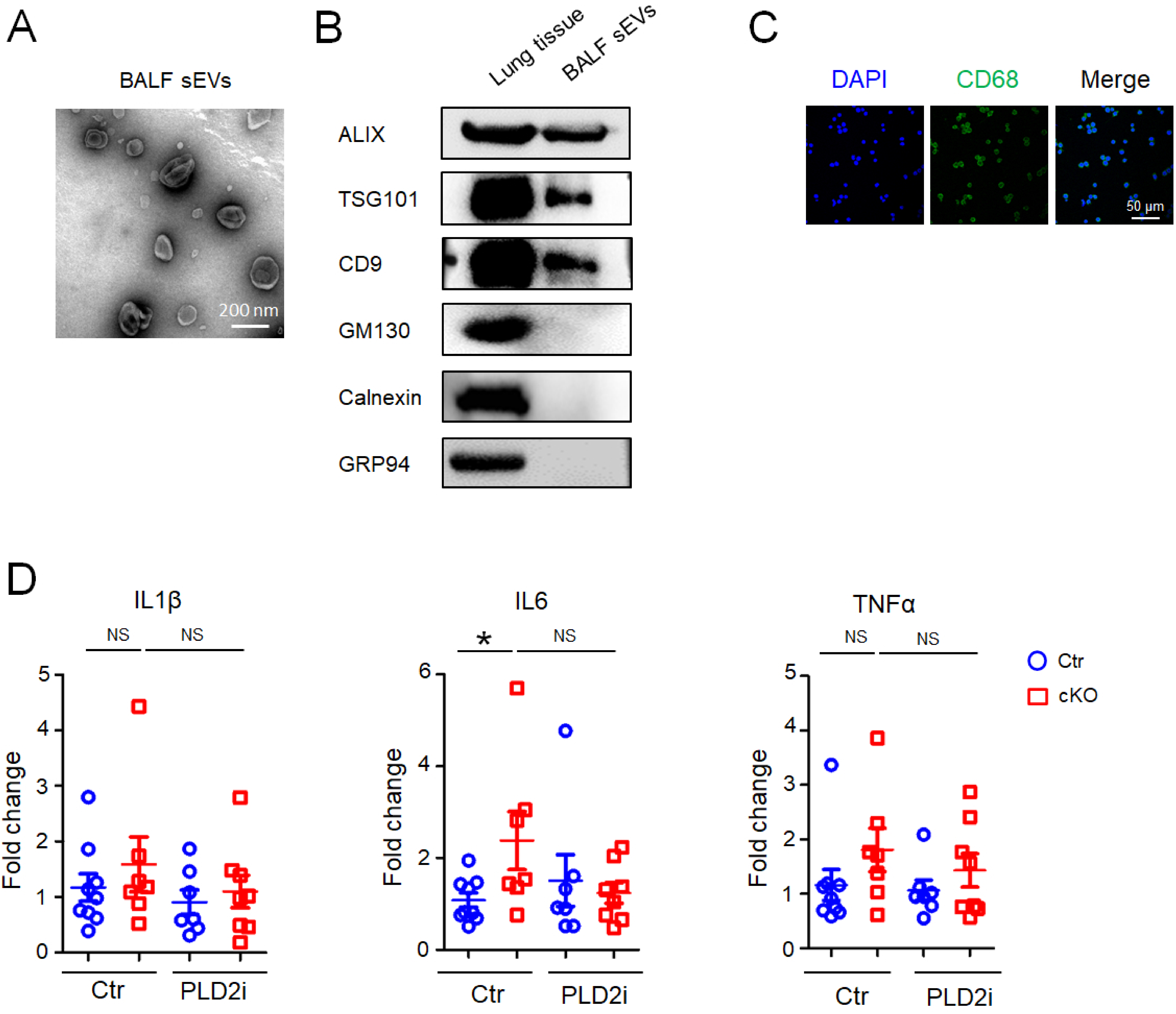
A
BALF sEVs
200 nm
B
Lung tissue
BALF sEVs
ALIX
TSG101
CD9
GM130
Calnexin
GRP94
C
DAPI
CD68
Merge
50 µm
D
IL1β
IL6
TNFα
NS
*
Fold change
Ctr
PLD2i
Ctr
cKO
